# Pyrazine-bridged polymetallic copper–iridium clusters

**DOI:** 10.1107/S2056989024007151

**Published:** 2024-07-27

**Authors:** Ben. J. Tickner, Richard Gammons, Adrian C. Whitwood, Simon B. Duckett

**Affiliations:** a Centre for Hyperpolarisation in Magnetic Resonance, University of York, Heslington, United Kingdom, YO10 5NY; b Department of Chemistry, University of York, Heslington, United Kingdom, YO10, 5DD; Vienna University of Technology, Austria

**Keywords:** crystal structure, clusters, polymetallic, heterometallic, Cu, Ir, pyrazine

## Abstract

Single crystals of the mol­ecular compound, {Cu_20_Ir_6_Cl_8_(C_21_H_24_N_2_)_6_(C_4_H_4_N_2_)_3_]·3.18CH_3_OH or [({Cu_10_Ir_3_}Cl_4_(IMes)_3_(pyrazine))_2_(pyrazine)]·3.18CH_3_OH [where IMes is 1,3-bis­(2,4,6-trimethylphen­yl)imidazol-2-yl­idene], with a unique heterometallic cluster have been prepared and the structure revealed using single-crystal X-ray diffraction. The mol­ecule is centrosymmetric with two {Cu_10_Ir_3_} cores bridged by a pyrazine ligand. The polymetallic cluster contains three stabilizing *N*-heterocyclic carbenes, four Cl ligands, and a non-bridging pyrazine ligand. Notably, the Cu—Ir core is arranged in an unusual shape containing 13 vertices, 22 faces, and 32 sides. The atoms within the trideca­metallic cluster are arranged in four planes, with 2, 4, 4, 3 metals in each plane. Ir atoms are present in alternate planes with an Ir atom featuring in the peripheral bimetallic plane, and two Ir atoms featuring on opposite sides of the non-adjacent tetra­metallic plane. The crystal contains two disordered methanol solvent mol­ecules with an additional region of non-modelled electron density corrected for using the SQUEEZE routine in *PLATON* [Spek (2015 ▸). *Acta Cryst.* C**71**, 9–18]. The given chemical formula and other crystal data do not take into account the unmodelled methanol solvent mol­ecule(s).

## Abstract 2

The title mol­ecule is centrosymmetric, with a pyrazine ligand bridging two {Cu_10_Ir_3_} cluster units that are arranged in an unusual shape containing 13 vertices, 22 faces, and 32 sides.

## 1. Chemical context

Polynuclear metallic clusters, particularly those featuring organic ligands, are highly important as they can appear as inter­mediates or decomposition products in many transition-metal-catalysed reactions. Metallic clusters can also exhibit properties between monometallic transition-metal complexes and higher order aggregates and nanoparticles (Tang & Zhao, 2020 ▸). Therefore, their synthesis, preparation, and analysis is highly important to advance current understanding on how such species can play a role in catalysis. Metal clusters based on Cu are particularly exciting as a wide range of Cu*_x_X_y_L_z_* clusters have been reported, where *X* is typically a halide or hydride, and *L* is a thio­ester, phosphine, or *N*-heterocycle (Harvey & Knorr, 2016 ▸; Dhayal *et al.*, 2016 ▸; Graham *et al.*, 2000 ▸; Liu & Astruc, 2018 ▸; Troyano *et al.*, 2021 ▸). There are also examples of heterometallic clusters containing Cu atoms mixed with a range of other transition metals such as Re, Fe, Ir, Os, Co, Mo, W, Ag, and Au (Sculfort & Braunstein, 2011 ▸; Croizat *et al.*, 2016 ▸; Hau *et al.*, 2016 ▸; Yip *et al.*, 2007 ▸; Gao *et al.*, 2024 ▸; Zhang *et al.*, 2023*a* ▸). These mixed metal clusters provide a unique example to explore metalophilic inter­actions (Sculfort & Braunstein, 2011 ▸) and often have novel spectroscopic properties (Yip *et al.*, 2007 ▸; Zhang *et al.*, 2023*a* ▸) or catalytic activity (Gao *et al.*, 2024 ▸; Zhang *et al.*, 2023*a* ▸), particularly as Cu complexes find many uses in carbon–carbon and carbon–heteroatom bond formation. To this end, we were able to grow single crystals of a novel heterometallic cluster compound containing two {Cu_10_Ir_3_} units bridged by a pyrazine ligand, which was examined using X-ray diffraction studies.
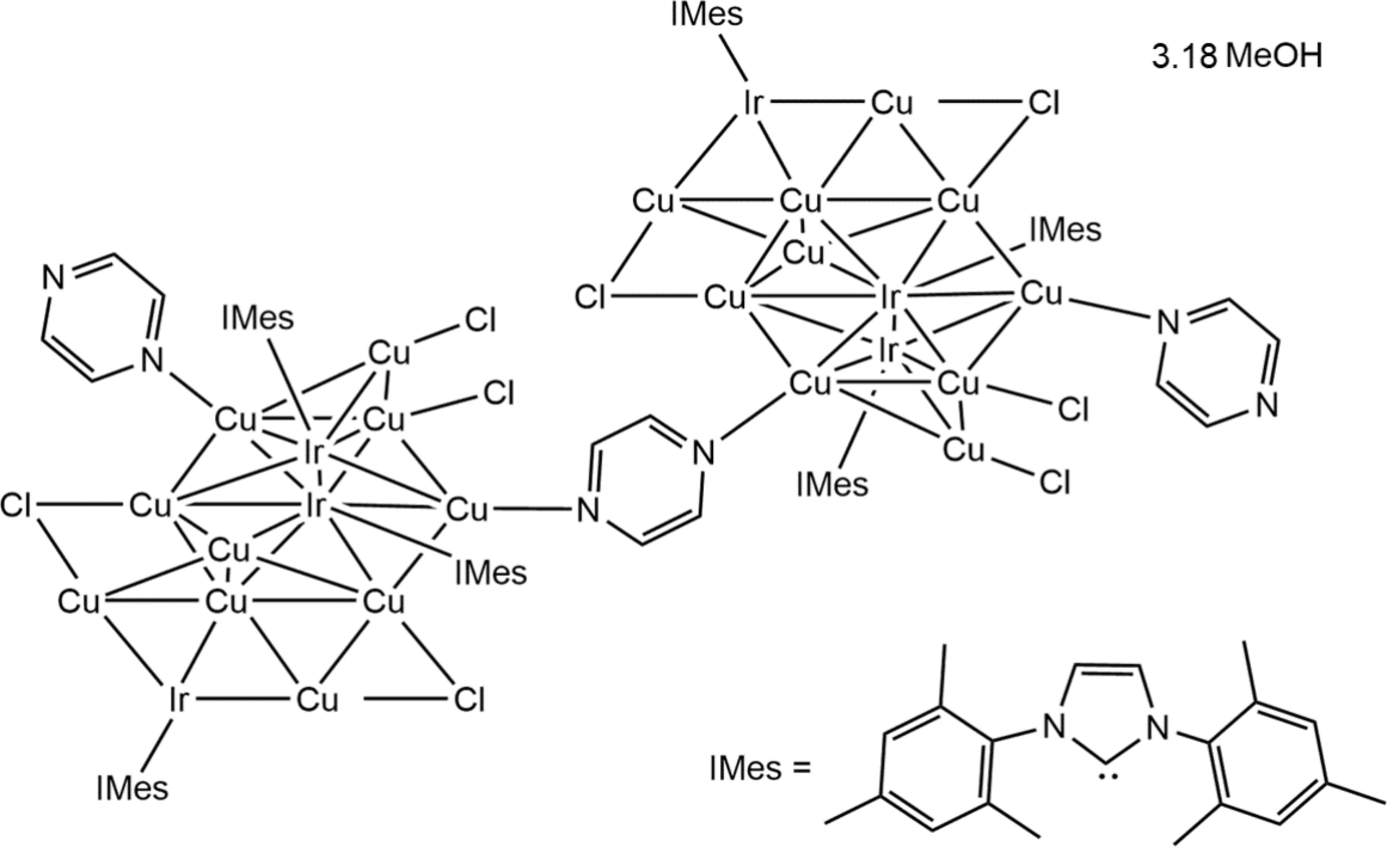


## 2. Structural commentary

**Figure 1:**
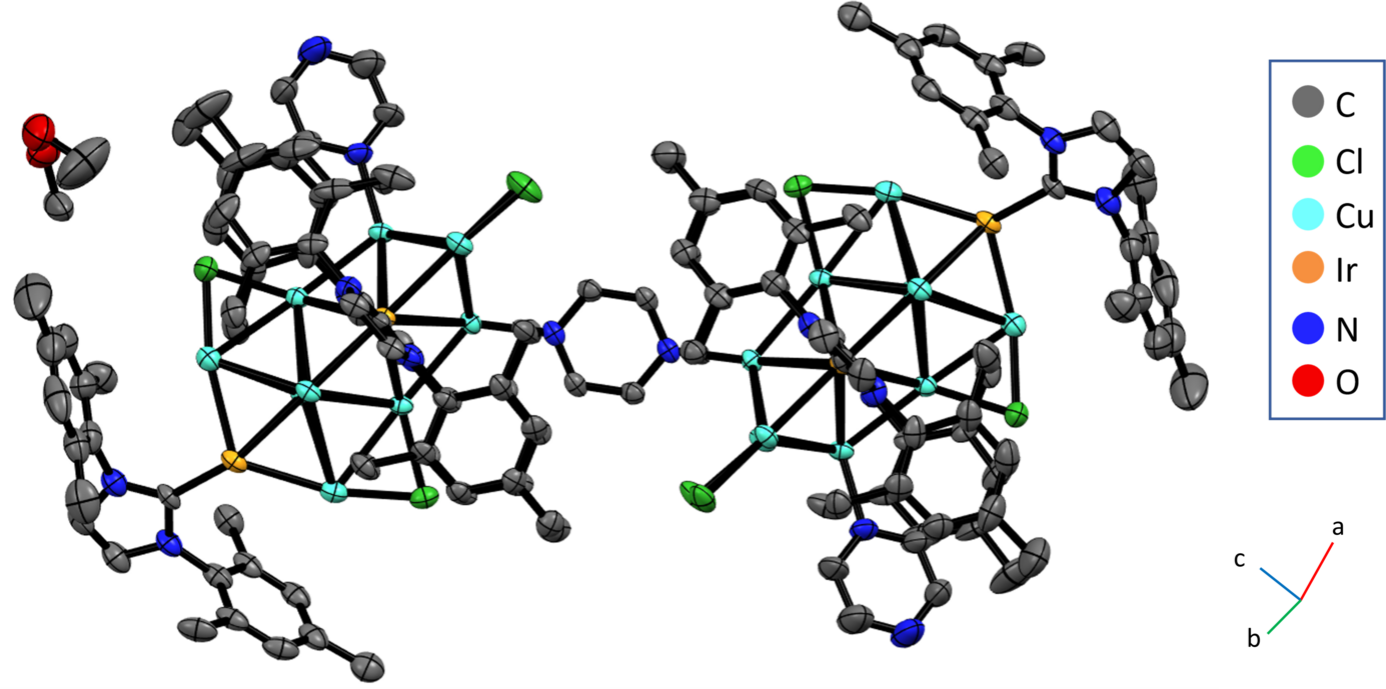
Mol­ecular structure of [(Cu_10_Ir_3_Cl_4_(IMes)_3_(pyrazine))_2_(pyrazine)]·3.18CH_3_OH, with displacement ellipsoids drawn at the 50% probability level. Hydrogen atoms were omitted for clarity.

**Figure 2:**
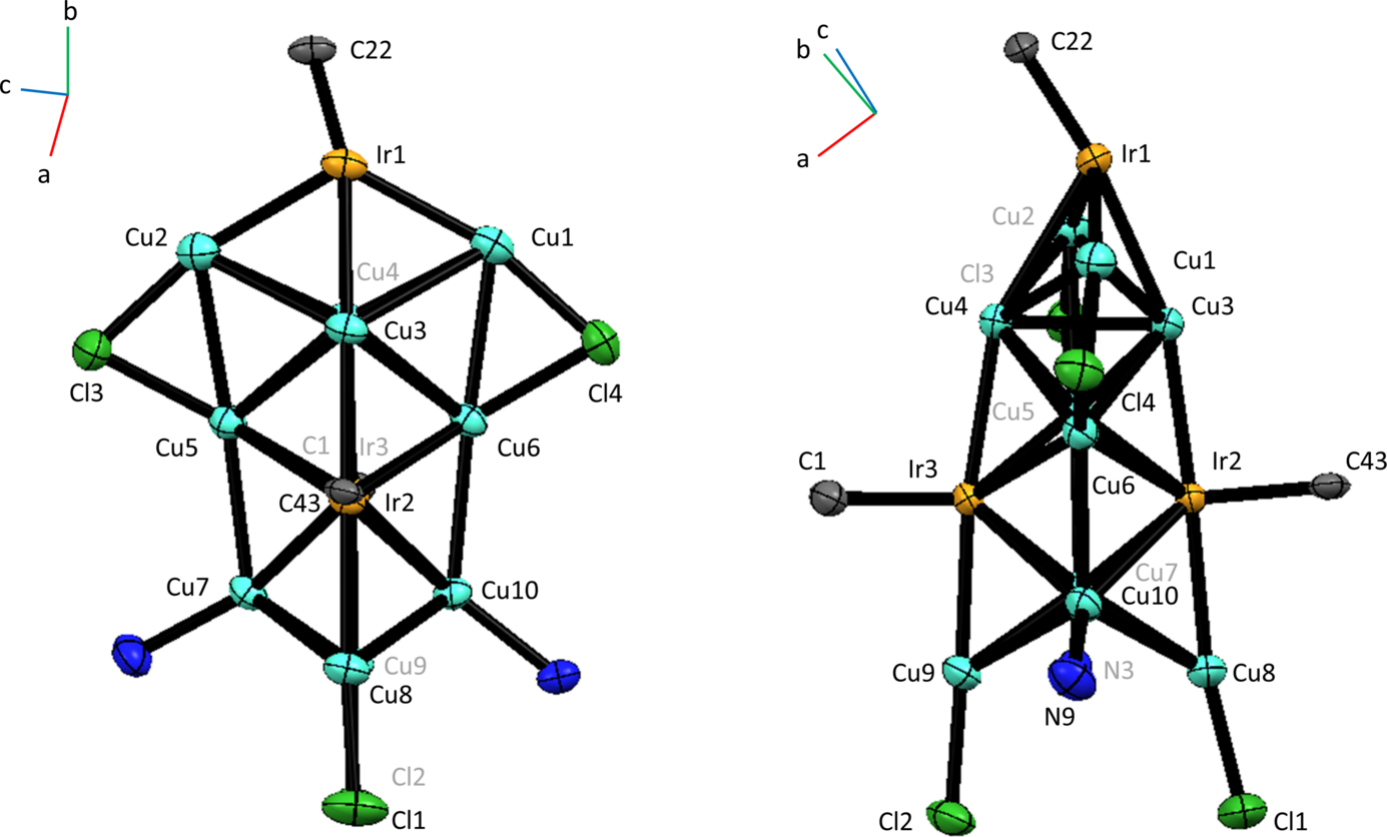
The trideca­metallic core of [(Cu_10_Ir_3_Cl_4_(IMes)_3_(pyrazine))_2_(pyrazine)]·3.18CH_3_OH, with displacement ellipsoids drawn at the 50% probability level. Note that only the donor atoms of the ligands attached to the polyatomic core are shown. The core is shown in two different orientations, rotated by 90° around the Ir1, Cu3, Cu4, Ir2, Ir3, Cu8, Cu9, Cu8, Cu9 plane. Atom labels marked in grey correspond to atoms hidden from view. The centrosymmetric compound contains two of these cores linked by a bridging pyrazine and therefore the two trideca­metallic units are equivalent by symmetry.

The solvated mol­ecular title compound [({Cu_10_Ir_3_}Cl_4_(IMes)_3_(pyrazine))_2_(pyrazine)]·3.18CH_3_OH (where IMes is 1,3-bis­(2,4,6-trimethyl-phen­yl)imidazol-2-yl­idene) is centrosymmetric and contains two trideca­metallic {Cu_10_Ir_3_} clusters, stabilised by four Cl ligands, three *N*-heterocyclic carbene (IMes) ligands, and a pyrazine ligand, with a bridging pyrazine mol­ecule linking two of these [{Cu_10_Ir_3_}Cl_4_(IMes)_3_(pyrazine)] units (Fig. 1 ▸). The {Cu_10_Ir_3_} cores are arranged in a geometry containing 13 vertices, 22 faces, and 32 sides with the atoms arranged in four planes with 2, 4, 4 and 3 metals in each plane (Fig. 2 ▸). The majority of the core consists of Cu atoms, with two existing as naked atoms with only inter­actions to adjacent Cu and Ir atoms. Of the remaining eight Cu sites, four are bonded to Cl ligands that bridge two Cu atoms across different atomic planes within the metallic core. Two of the three Cu atoms in a peripheral plane are bonded to terminal Cl ligands, with the third ligated to a terminal pyrazine mol­ecule. Inter­estingly, a bridging pyrazine ligand is bonded to a Cu atom in a tetra­metallic plane and provides a link to another [{Cu_10_Ir_3_}Cl_4_(NHC)_3_(pyrazine)] unit, with the whole mol­ecule having a centre of inversion in the middle of the bridging pyrazine ring. Ir atoms are located in alternate planes with an Ir atom featuring in the peripheral bimetallic plane, and two Ir atoms featuring on opposite sides of the non-adjacent tetra­metallic plane. This arrangement is likely a consequence of the bulky carbene ligand attached to Ir. All 18 Cu—Cu distances range from 2.4916 (18) to 3.0417 (18) Å. All but three of these distances are shorter than the sum of the van der Waals radii of Cu (2.80 Å), and most are close to the sum of the Cu atomic radii (2.556 Å), which suggests strong metalophilic inter­actions within the cluster (Sculfort & Braunstein, 2011 ▸). There appear to be no significant differences between the Cu—Cu and Ir—Cu bond lengths in the structure (2.66 ± 0.13 Å, *n* = 18 and 2.62 ± 0.07 Å, *n* = 16).

## 3. Supra­molecular features

**Figure 3:**
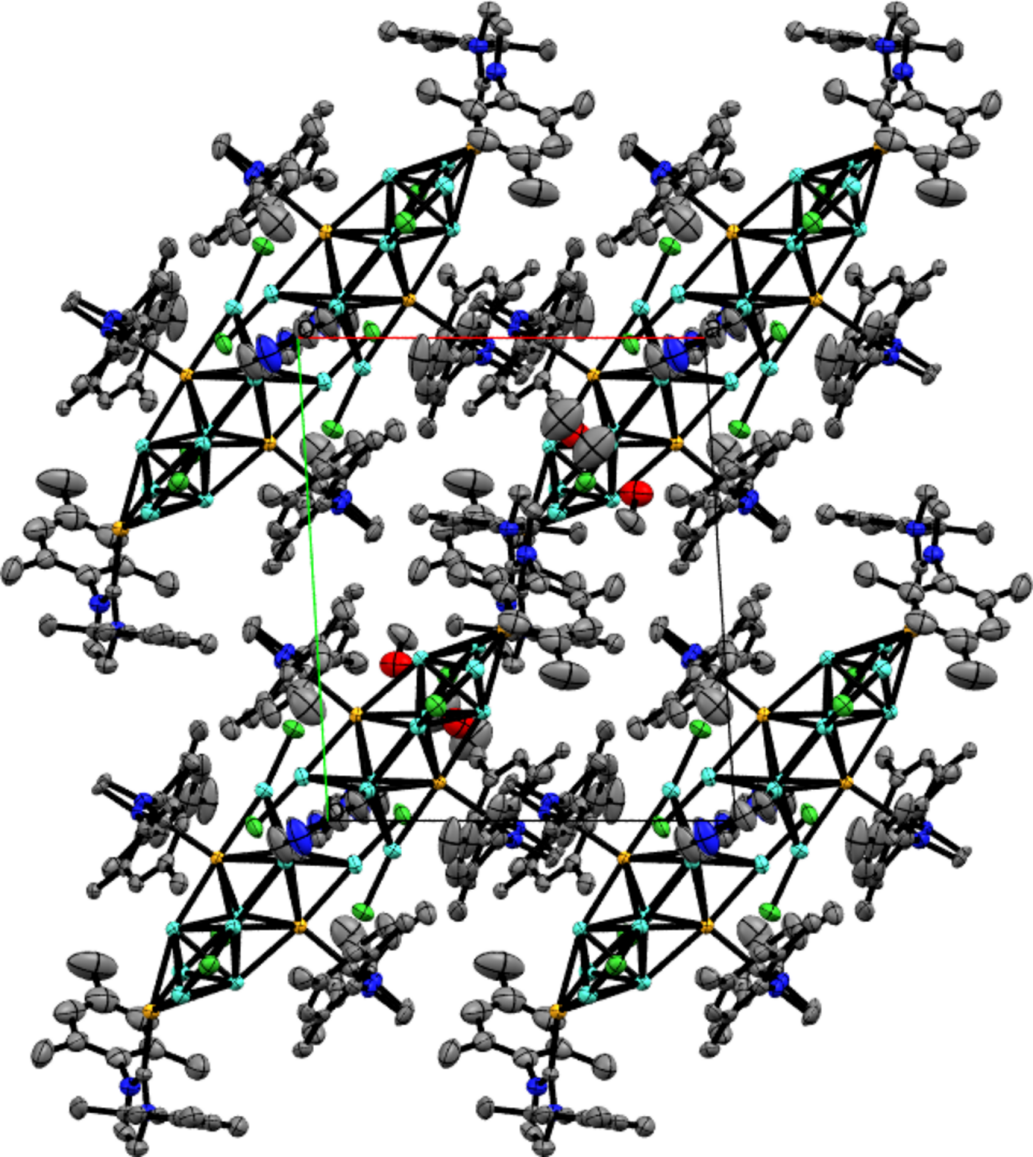
Crystal packing of [(Cu_10_Ir_3_Cl_4_(IMes)_3_(pyrazine))_2_(pyrazine)]·3.18CH_3_OH shown in a view along the *c* axis. Displacement ellipsoids are drawn at the 50% probability level and hydrogen atoms were omitted for clarity.

The methanol solvent mol­ecules clearly fill voids left by the packing of [({Cu_10_Ir_3_}Cl_4_(IMes)_3_(pyrazine))_2_(pyrazine)] as the shortest inter­actions are between methanol and the three terminal CH_3_ groups of the IMes ligand. Long-range inter­actions between the mol­ecules of [({Cu_10_Ir_3_}Cl_4_(IMes)_3_(pyrazine))_2_(pyrazine)] involve the non-bridging pyrazine ligands on adjacent mol­ecules, with the shortest 2.327 Å inter­action between the two H65 atoms, and a 2.483 Å inter­action between the free pyrazine N4 and the H65 atom of a non-bridging pyrazine ligand on a neighbouring mol­ecule. This suggests that the pyrazine ligand is important in both linking the two trideca­metallic cores, and also packing the crystals together, which is unsurprising given its role in the formation of higher order polymers and metal–organic frameworks (Silva *et al.*, 2023 ▸; Zhang *et al.*, 2023*b* ▸; Kawamura *et al.*, 2017 ▸). Long-range inter­actions between IMes ligands of different mol­ecules are also important with distances of 2.377 Å and 2.383 Å between pairs of *ortho* CH_3_ and *para* CH_3_ groups on the mesityl rings of adjacent mol­ecules (H19*B*/H41*C* and H20*B* and H42*B*). The crystal packing is shown in Fig. 3 ▸. The hydroxyl hydrogen atom (H2*A*) of the partially occupied methanol solvent mol­ecule is hydrogen-bonded to the oxygen atom of the other disordered methanol mol­ecule (Table 1 ▸). It should be noted, however, that the hydrogen atom is placed using a riding model as allowing free refinement of its coordinates gave an unfeasible result. The hydroxyl H atoms of the other methanol mol­ecules are likely to be hydrogen-bonded to other highly disordered solvent mol­ecules that were not modelled using the solvent mask (see *Refinement*).

## 4. Database survey

A search of the Cambridge Structure Database (CSD, Version 5.45, update November 2023; Groom *et al.*, 2016 ▸) did not reveal any comparable compounds with trideca­metallic polymetallic clusters. A few crystal structures for penta­tomic Cu–Ir clusters have been reported, but these contain cores with a trigonal–bipyramidal shape with either Cu_3_Ir_2_*L_x_* (Rhodes *et al.*, 1985 ▸) or Ir_4_Cu*L_x_* (Adams *et al.*, 2013 ▸) arrangements. Reported Cu—Ir distances are between 2.663 and 2.79 Å, which are generally longer than those in the cluster presented here [2.5227 (15) to 2.7478 (13) Å]. The short Cu—Ir distances suggest strong metal–metal inter­actions, and could indicate Cu=Ir bonds (Rhodes *et al.*, 1985 ▸). There are many more examples of homometallic Cu clusters in the database, an analysis of 35 of these Cu—Cu bond lengths revealed an average inter­metallic distance of 2.95 ± 0.25 Å (mean ± 1 standard deviation), which is consistent with the inter Cu distances in the structure reported here (2.66 ± 0.13 Å), albeit slightly longer (Johnsson *et al.*, 2000 ▸; Rao *et al.*, 1983 ▸; Baumgartner *et al.*, 1990 ▸). Similar to short Cu—Ir distances, this suggests that the Cu—Cu inter­actions are also strong and metalophillic.

## 5. Synthesis and crystallization

The pyrazine-bridged polymetallic Cu–Ir cluster compound was prepared by reaction of [Ir(Cl)(COD)(IMes)] (2.20 mg) [COD is *cis*,*cis*-1,5-cyclo­octa­diene and IMes is 1,3-bis­(2,4,6-trimethyl-phen­yl)imidazol-2-yl­idene] with pyrazine (2.52 mg) and H_2_ (3 bar) in methanol-*d*_4_ (0.6 ml) for 3–4 h at 298 K in a 5 mm NMR tube with a J. Youngs tap. At this point the pressure was released by opening the lid and Cu(OAc)_2_ (3.76 mg) in methanol-*d*_4_ (0.1 ml) was added to the solution. After being left for 1 h at room temperature the solution was cooled to 278 K in a refrigerator for several weeks to form single crystals, which were found by X-ray diffraction to be the title compound.

## 6. Refinement

Crystal data, data collection and structure refinement details are summarized in Table 2 ▸. All hydrogen atoms were placed using a riding model. The crystal contains disordered methanol solvent mol­ecules. One methanol mol­ecule was modelled over two sets of sites (C70, C72) with a common oxygen site (O1) in a refined ratio of 0.60:0.40 (3). Another methanol mol­ecule (C71, O2) was modelled as partially occupied with a refined occupancy of 0.59 (2). There was additional solvent present, but its associated electron density was difficult to model by using discrete atoms. Therefore the SQUEEZE routine (Spek, 2015 ▸) in *PLATON* (Spek, 2020 ▸) was used to remove the contribution of the electron density in the corresponding solvent region from the intensity data. A void with a volume of 430 Å^3^ was predicted containing 66 electrons per unit cell. This would be equivalent to 3.67 methanol mol­ecules. The given chemical formula and other crystal data do not take into account the unmodelled methanol solvent mol­ecule(s). The final structure model contains high residual electron density due to unresolved effects of the crystal having a minor twin present. Attempts to model this as two non-merohedral components were unsuccessful.

## Supplementary Material

Crystal structure: contains datablock(s) I. DOI: 10.1107/S2056989024007151/wm5728sup1.cif

Structure factors: contains datablock(s) I. DOI: 10.1107/S2056989024007151/wm5728Isup2.hkl

Chemical Connectivity (.mol) files. DOI: 10.1107/S2056989024007151/wm5728sup3.mol

CCDC reference: 2364376

Additional supporting information:  crystallographic information; 3D view; checkCIF report

## Figures and Tables

**Table 1:** Hydrogen-bond geometry (Å, °)

*D*—H⋯*A*	*D*—H	H⋯*A*	*D*⋯*A*	*D*—H⋯*A*
O2—H2*A*⋯O1	0.84	1.87	2.62 (2)	148

**Table 2:** Experimental details

Crystal data	
Chemical formula	{Cu_20_Ir_6_Cl_8_(C_21_H_24_N_2_)_6_(C_4_H_4_N_2_)_3_]·3.18CH_4_O
*M*_r_	4876.63
Crystal system, space group	Triclinic, *P* 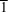
Temperature (K)	111
*a*, *b*, *c* (Å)	12.5708 (3), 14.8757 (5), 23.1383 (7)
α, β, γ (°)	84.392 (3), 82.291 (2), 85.686 (2)
*V* (Å^3^)	4258.8 (2)
*Z*	1
Radiation type	Cu *K*α
μ (mm^−1^)	12.93
Crystal size (mm)	0.15 × 0.12 × 0.07

Data collection	
Diffractometer	SuperNova, Dual, Cu at home/near, HyPix
Absorption correction	Analytical [*CrysAlis PRO* (Rigaku OD, 2024 ▸) using a multifaceted crystal model based on expressions derived by Clark & Reid (1995 ▸)]
*T*_min_, *T*_max_	0.222, 0.519
No. of measured, independent and observed [*I* > 2σ(*I*)] reflections	45533, 15184, 13473
*R*_int_	0.046
(sin θ/λ)_max_ (Å^−1^)	0.597

Refinement	
*R*[*F*^2^ > 2σ(*F*^2^)], *wR*(*F*^2^), *S*	0.054, 0.134, 1.08
No. of reflections	15184
No. of parameters	919
H-atom treatment	H-atom parameters constrained
Δρ_max_, Δρ_min_ (e Å^−3^)	3.01, −2.57

Computer programs: *CrysAlis PRO* (Rigaku OD, 2024 ▸), *SHELXT* (Sheldrick, 2015*a* ▸), *SHELXL* (Sheldrick, 2015*b* ▸) and *OLEX2* (Dolomanov *et al.*, 2009 ▸).

## supplementary crystallographic information

### Ben Tickner BJT004 Crystal data

**Table:**

{Cu_20_Ir_6_Cl_8_(C_21_H_24_N_2_)_6_(C_4_H_4_N_2_)_3_]·3.18CH_4_O	*Z* = 1
*M**_r_* = 4876.63	*F*(000) = 2345
Triclinic, *P*1	*D*_x_ = 1.901 Mg m^−^^3^
*a* = 12.5708 (3) Å	Cu *K*α radiation, λ = 1.54184 Å
*b* = 14.8757 (5) Å	Cell parameters from 20996 reflections
*c* = 23.1383 (7) Å	θ = 3.6–75.9°
α = 84.392 (3)°	µ = 12.93 mm^−^^1^
β = 82.291 (2)°	*T* = 111 K
γ = 85.686 (2)°	Block, clear orange
*V* = 4258.8 (2) Å^3^	0.15 × 0.12 × 0.07 mm

### Ben Tickner BJT004 Data collection

**Table:**

SuperNova, Dual, Cu at home/near, HyPix diffractometer	15184 independent reflections
Radiation source: micro-focus sealed X-ray tube, SuperNova (Cu) X-ray Source	13473 reflections with *I* > 2σ(*I*)
Mirror monochromator	*R*_int_ = 0.046
Detector resolution: 10.0000 pixels mm^-1^	θ_max_ = 67.1°, θ_min_ = 3.6°
ω scans	*h* = −13→15
Absorption correction: analytical [CrysAlisPro (Rigaku OD, 2024) using a multifaceted crystal model based on expressions derived by Clark & Reid (1995)]	*k* = −17→17
*T*_min_ = 0.222, *T*_max_ = 0.519	*l* = −27→25
45533 measured reflections	

### Ben Tickner BJT004 Refinement

**Table:**

Refinement on *F*^2^	Primary atom site location: dual
Least-squares matrix: full	Hydrogen site location: inferred from neighbouring sites
*R*[*F*^2^ > 2σ(*F*^2^)] = 0.054	H-atom parameters constrained
*wR*(*F*^2^) = 0.134	*w* = 1/[σ^2^(*F*_o_^2^) + (0.0427*P*)^2^ + 57.022*P*] where *P* = (*F*_o_^2^ + 2*F*_c_^2^)/3
*S* = 1.08	(Δ/σ)_max_ = 0.001
15184 reflections	Δρ_max_ = 3.01 e Å^−^^3^
919 parameters	Δρ_min_ = −2.56 e Å^−^^3^
0 restraints	

### Ben Tickner BJT004 Special details

**Table:**

Geometry. All esds (except the esd in the dihedral angle between two l.s. planes) are estimated using the full covariance matrix. The cell esds are taken into account individually in the estimation of esds in distances, angles and torsion angles; correlations between esds in cell parameters are only used when they are defined by crystal symmetry. An approximate (isotropic) treatment of cell esds is used for estimating esds involving l.s. planes.
Refinement. The high residual density is due to unresolved effects of the crystal having a minor twin present. Attempts to model this as two non-merohedral components were unsuccessful.The solvent was disordered and modelled as follows: One methanol was modelled in two positions with a common oxygen site in a refined ratio of 0.60:0.40 (3). Another methanol was partially occupied with a refined occupancy of 0.59 (2). There was additional solvent which was too disordered to model using discrete atoms. Therefore a solvent mask was used which predicted a void with a volume of 430 cubic angstroms containing 66 electrons per unit cell. This would be equivalent to 3.67 methanols.

### Ben Tickner BJT004 Fractional atomic coordinates and isotropic or equivalent isotropic displacement parameters (Å^2^)

**Table:**

	*x*	*y*	*z*	*U*_iso_*/*U*_eq_	Occ. (<1)
C1	1.0318 (7)	0.3061 (6)	0.6817 (4)	0.035 (2)	
C2	1.1554 (9)	0.4028 (7)	0.6378 (5)	0.048 (3)	
H2	1.197662	0.438919	0.608630	0.058*	
C3	1.1621 (8)	0.3952 (8)	0.6941 (5)	0.050 (3)	
H3	1.209564	0.425577	0.713107	0.060*	
C4	1.0741 (8)	0.3107 (7)	0.7834 (5)	0.044 (2)	
C5	0.9968 (8)	0.3610 (8)	0.8182 (5)	0.047 (3)	
C6	0.9884 (10)	0.3365 (10)	0.8779 (6)	0.064 (3)	
H6	0.939332	0.370857	0.903401	0.077*	
C7	1.0488 (12)	0.2642 (10)	0.9020 (5)	0.067 (4)	
C8	1.1257 (10)	0.2196 (10)	0.8657 (5)	0.059 (3)	
H8	1.170636	0.172291	0.881957	0.071*	
C9	1.1393 (9)	0.2419 (8)	0.8060 (5)	0.049 (3)	
C10	1.2261 (9)	0.1914 (8)	0.7665 (5)	0.051 (3)	
H10A	1.255207	0.137970	0.788869	0.076*	
H10B	1.284078	0.231426	0.751748	0.076*	
H10C	1.194777	0.172379	0.733399	0.076*	
C11	1.0327 (18)	0.2366 (15)	0.9680 (7)	0.111 (7)	
H11A	1.052659	0.285620	0.988977	0.167*	
H11B	1.078120	0.181718	0.976591	0.167*	
H11C	0.957085	0.224777	0.980575	0.167*	
C12	0.9306 (10)	0.4388 (9)	0.7939 (6)	0.061 (3)	
H12A	0.861173	0.418332	0.787373	0.091*	
H12B	0.968202	0.463813	0.756583	0.091*	
H12C	0.918737	0.485468	0.821509	0.091*	
C13	1.0378 (7)	0.3438 (6)	0.5732 (4)	0.036 (2)	
C14	0.9542 (8)	0.4051 (6)	0.5584 (4)	0.036 (2)	
C15	0.9159 (8)	0.3985 (7)	0.5062 (5)	0.045 (3)	
H15	0.858738	0.439625	0.495576	0.054*	
C16	0.9574 (8)	0.3347 (7)	0.4685 (5)	0.042 (2)	
C17	1.0410 (8)	0.2763 (6)	0.4834 (4)	0.038 (2)	
H17	1.070598	0.232705	0.457394	0.045*	
C18	1.0844 (7)	0.2791 (6)	0.5365 (4)	0.033 (2)	
C19	1.1739 (8)	0.2120 (7)	0.5515 (4)	0.041 (2)	
H19A	1.193399	0.222273	0.589841	0.061*	
H19B	1.236496	0.219280	0.521718	0.061*	
H19C	1.150074	0.150453	0.552869	0.061*	
C20	0.9080 (11)	0.3269 (9)	0.4127 (6)	0.060 (3)	
H20A	0.915440	0.383296	0.387246	0.090*	
H20B	0.831607	0.315697	0.422700	0.090*	
H20C	0.945217	0.276554	0.392219	0.090*	
C21	0.9017 (9)	0.4742 (6)	0.5987 (5)	0.046 (3)	
H21A	0.945474	0.477558	0.630305	0.070*	
H21B	0.829769	0.456311	0.615340	0.070*	
H21C	0.895905	0.533535	0.576465	0.070*	
C22	0.5071 (7)	0.5250 (6)	0.7576 (4)	0.033 (2)	
C23	0.4786 (9)	0.6775 (7)	0.7541 (6)	0.053 (3)	
H23	0.475773	0.739295	0.739084	0.063*	
C24	0.4520 (10)	0.6458 (8)	0.8099 (6)	0.055 (3)	
H24	0.425422	0.680157	0.841955	0.066*	
C25	0.4475 (11)	0.4919 (8)	0.8646 (5)	0.056 (3)	
C26	0.3482 (10)	0.4575 (8)	0.8779 (6)	0.056 (3)	
C27	0.3347 (16)	0.3967 (10)	0.9276 (8)	0.093 (6)	
H27	0.267293	0.370551	0.937888	0.111*	
C28	0.4164 (18)	0.3720 (11)	0.9634 (7)	0.088 (5)	
C29	0.5119 (15)	0.4109 (10)	0.9491 (6)	0.078 (4)	
H29	0.566822	0.395811	0.973570	0.094*	
C30	0.5324 (11)	0.4720 (9)	0.9001 (5)	0.058 (3)	
C31	0.6401 (11)	0.5102 (10)	0.8842 (7)	0.071 (4)	
H31A	0.671998	0.516138	0.919895	0.107*	
H31B	0.631953	0.569782	0.862576	0.107*	
H31C	0.687115	0.469535	0.859602	0.107*	
C32	0.397 (2)	0.3010 (14)	1.0156 (9)	0.150 (12)	
H32A	0.459982	0.293309	1.036595	0.226*	
H32B	0.383977	0.243251	1.001480	0.226*	
H32C	0.333790	0.321082	1.042001	0.226*	
C33	0.2617 (12)	0.4824 (11)	0.8403 (8)	0.082 (5)	
H33A	0.289867	0.474198	0.799465	0.123*	
H33B	0.236542	0.545832	0.843845	0.123*	
H33C	0.201697	0.443617	0.852873	0.123*	
C34	0.5373 (8)	0.6118 (6)	0.6598 (5)	0.042 (2)	
C35	0.4557 (8)	0.6070 (7)	0.6251 (5)	0.045 (3)	
C36	0.4837 (9)	0.6099 (7)	0.5651 (6)	0.049 (3)	
H36	0.429176	0.606813	0.540731	0.059*	
C37	0.5893 (10)	0.6173 (7)	0.5396 (5)	0.050 (3)	
C38	0.6675 (9)	0.6261 (6)	0.5761 (5)	0.046 (3)	
H38	0.739490	0.633667	0.558659	0.056*	
C39	0.6451 (8)	0.6244 (6)	0.6357 (5)	0.041 (2)	
C40	0.7302 (9)	0.6340 (8)	0.6749 (5)	0.051 (3)	
H40A	0.737606	0.578455	0.700968	0.076*	
H40B	0.708924	0.685306	0.698412	0.076*	
H40C	0.799155	0.644472	0.650800	0.076*	
C41	0.6179 (11)	0.6145 (8)	0.4747 (6)	0.060 (3)	
H41A	0.557918	0.642068	0.454760	0.090*	
H41B	0.632283	0.551431	0.465523	0.090*	
H41C	0.682175	0.648037	0.461501	0.090*	
C42	0.3415 (9)	0.5954 (8)	0.6536 (6)	0.057 (3)	
H42A	0.340175	0.542898	0.682740	0.085*	
H42B	0.296246	0.585810	0.623645	0.085*	
H42C	0.314001	0.649819	0.672788	0.085*	
C43	0.5943 (7)	0.0014 (6)	0.7238 (4)	0.0305 (19)	
C44	0.4553 (7)	−0.0818 (6)	0.7667 (5)	0.035 (2)	
H44	0.408741	−0.114083	0.795879	0.042*	
C45	0.4444 (7)	−0.0675 (5)	0.7096 (4)	0.0320 (19)	
H45	0.389201	−0.087726	0.690686	0.038*	
C46	0.5480 (7)	0.0158 (6)	0.6220 (4)	0.0295 (18)	
C47	0.4926 (7)	0.0967 (6)	0.6037 (4)	0.0298 (18)	
C48	0.5177 (7)	0.1302 (6)	0.5460 (4)	0.0322 (19)	
H48	0.481170	0.184933	0.532557	0.039*	
C49	0.5936 (8)	0.0878 (6)	0.5071 (4)	0.036 (2)	
C50	0.6413 (7)	0.0053 (7)	0.5266 (4)	0.036 (2)	
H50	0.691616	−0.025935	0.499970	0.043*	
C51	0.6184 (8)	−0.0330 (7)	0.5833 (4)	0.038 (2)	
C52	0.6711 (9)	−0.1237 (7)	0.6023 (5)	0.043 (2)	
H52A	0.651164	−0.137681	0.644496	0.064*	
H52B	0.749424	−0.121611	0.593771	0.064*	
H52C	0.646965	−0.170613	0.581079	0.064*	
C53	0.6235 (8)	0.1308 (7)	0.4459 (4)	0.042 (2)	
H53A	0.666502	0.182602	0.447360	0.064*	
H53B	0.557915	0.151384	0.428601	0.064*	
H53C	0.665387	0.086350	0.422088	0.064*	
C54	0.4130 (7)	0.1455 (6)	0.6456 (4)	0.0327 (19)	
H54A	0.400305	0.208239	0.629448	0.039*	
H54B	0.441272	0.144947	0.683067	0.039*	
H54C	0.345150	0.115406	0.651616	0.039*	
C55	0.5841 (8)	−0.0404 (7)	0.8314 (4)	0.038 (2)	
C56	0.6601 (8)	−0.1098 (8)	0.8483 (5)	0.051 (3)	
C57	0.6933 (10)	−0.1070 (11)	0.9018 (6)	0.069 (4)	
H57	0.742674	−0.153961	0.914412	0.082*	
C58	0.6599 (11)	−0.0406 (13)	0.9385 (6)	0.077 (5)	
C59	0.5836 (10)	0.0241 (10)	0.9211 (5)	0.062 (3)	
H59	0.556086	0.068366	0.947083	0.074*	
C60	0.5452 (8)	0.0276 (8)	0.8679 (4)	0.043 (2)	
C61	0.4611 (9)	0.0975 (7)	0.8507 (5)	0.047 (3)	
H61A	0.390286	0.072385	0.859379	0.070*	
H61B	0.475601	0.115596	0.808619	0.070*	
H61C	0.462246	0.150389	0.872649	0.070*	
C62	0.7038 (13)	−0.0410 (17)	0.9968 (6)	0.110 (8)	
H62A	0.753787	−0.094116	1.001953	0.165*	
H62B	0.644139	−0.043313	1.028752	0.165*	
H62C	0.741609	0.014128	0.997153	0.165*	
C63	0.6958 (9)	−0.1866 (8)	0.8097 (6)	0.062 (3)	
H63A	0.746911	−0.164800	0.776410	0.093*	
H63B	0.633048	−0.207639	0.795326	0.093*	
H63C	0.730553	−0.236625	0.832544	0.093*	
C64	0.9285 (10)	0.0325 (10)	0.8800 (5)	0.061 (3)	
H64	0.860623	0.065569	0.883887	0.073*	
C65	0.9789 (11)	0.0104 (10)	0.9287 (5)	0.066 (4)	
H65	0.944111	0.027837	0.965277	0.080*	
C66	1.1209 (14)	−0.0494 (14)	0.8739 (7)	0.094 (6)	
H66	1.191030	−0.078550	0.870261	0.112*	
C67	1.0749 (11)	−0.0257 (11)	0.8246 (6)	0.073 (4)	
H67	1.115069	−0.033749	0.787502	0.087*	
C68	0.9250 (7)	0.0683 (6)	0.5015 (4)	0.0310 (19)	
H68	0.871317	0.116840	0.501075	0.037*	
C69	0.9720 (7)	0.0375 (6)	0.4489 (4)	0.034 (2)	
H69	0.950812	0.065952	0.413353	0.041*	
Cl1	0.9288 (2)	−0.18962 (18)	0.68466 (16)	0.0611 (8)	
Cl2	1.18296 (19)	−0.01264 (18)	0.66583 (12)	0.0474 (6)	
Cl3	0.7183 (2)	0.23791 (17)	0.87864 (10)	0.0435 (5)	
Cl4	0.6845 (2)	0.29938 (16)	0.55228 (10)	0.0400 (5)	
Cu1	0.60976 (12)	0.36171 (9)	0.63001 (6)	0.0385 (3)	
Cu2	0.61523 (12)	0.31363 (9)	0.81964 (6)	0.0388 (3)	
Cu3	0.60371 (10)	0.22542 (8)	0.72350 (6)	0.0309 (3)	
Cu4	0.74725 (10)	0.33490 (8)	0.71204 (6)	0.0314 (3)	
Cu5	0.76541 (10)	0.19426 (9)	0.78596 (6)	0.0306 (3)	
Cu6	0.74921 (10)	0.22104 (8)	0.63161 (5)	0.0295 (3)	
Cu7	0.90003 (10)	0.06243 (9)	0.75911 (6)	0.0314 (3)	
Cu8	0.84697 (11)	−0.06169 (9)	0.69873 (6)	0.0360 (3)	
Cu9	1.05763 (10)	0.09077 (9)	0.67851 (6)	0.0354 (3)	
Cu10	0.88794 (10)	0.08323 (8)	0.62802 (5)	0.0291 (3)	
Ir1	0.53490 (3)	0.39559 (3)	0.73285 (2)	0.03402 (11)	
Ir2	0.72239 (3)	0.07873 (2)	0.71117 (2)	0.02503 (10)	
Ir3	0.91336 (3)	0.21791 (2)	0.69321 (2)	0.02633 (10)	
N1	1.0875 (6)	0.3350 (6)	0.7213 (4)	0.0393 (19)	
N2	1.0739 (6)	0.3475 (5)	0.6283 (4)	0.0358 (17)	
N3	0.9707 (7)	0.0097 (6)	0.8279 (4)	0.044 (2)	
N4	1.0731 (10)	−0.0340 (11)	0.9270 (5)	0.084 (4)	
N5	0.4716 (7)	0.5509 (6)	0.8115 (4)	0.045 (2)	
N6	0.5108 (7)	0.6043 (5)	0.7225 (4)	0.0420 (19)	
N7	0.5481 (6)	−0.0404 (5)	0.7756 (3)	0.0339 (17)	
N8	0.5311 (5)	−0.0165 (5)	0.6833 (3)	0.0283 (15)	
N9	0.9528 (6)	0.0319 (5)	0.5529 (3)	0.0306 (16)	
O1	0.6658 (10)	0.2014 (8)	1.1082 (5)	0.093 (4)	
H1A	0.631550	0.174657	1.137841	0.140*	0.60 (3)
H1B	0.713511	0.172916	1.126141	0.140*	0.40 (3)
C71	0.789 (2)	0.3806 (18)	1.0273 (10)	0.079 (9)	0.59 (2)
H71A	0.841724	0.365036	0.994000	0.119*	0.59 (2)
H71B	0.795794	0.443441	1.034988	0.119*	0.59 (2)
H71C	0.716331	0.373815	1.018157	0.119*	0.59 (2)
O2	0.8081 (17)	0.3229 (12)	1.0769 (7)	0.079 (6)	0.59 (2)
H2A	0.777651	0.274561	1.076662	0.119*	0.59 (2)
C70	0.637 (4)	0.169 (3)	1.0558 (15)	0.142 (14)	0.60 (3)
H70A	0.687774	0.189291	1.022099	0.213*	0.60 (3)
H70B	0.563992	0.193702	1.049769	0.213*	0.60 (3)
H70C	0.638408	0.102980	1.060148	0.213*	0.60 (3)
C72	0.711 (6)	0.227 (5)	1.047 (2)	0.142 (14)	0.40 (3)
H72A	0.661064	0.213192	1.020360	0.213*	0.40 (3)
H72B	0.780038	0.192371	1.037713	0.213*	0.40 (3)
H72C	0.722683	0.291715	1.041743	0.213*	0.40 (3)

### Ben Tickner BJT004 Atomic displacement parameters (Å^2^)

**Table:**

	*U*^11^	*U*^22^	*U*^33^	*U*^12^	*U*^13^	*U*^23^
C1	0.029 (5)	0.034 (5)	0.042 (5)	0.001 (4)	−0.006 (4)	−0.007 (4)
C2	0.036 (6)	0.045 (6)	0.064 (7)	−0.024 (5)	0.004 (5)	−0.005 (5)
C3	0.024 (5)	0.071 (8)	0.057 (7)	−0.014 (5)	0.001 (4)	−0.016 (6)
C4	0.038 (6)	0.050 (6)	0.048 (6)	−0.014 (5)	0.000 (4)	−0.022 (5)
C5	0.029 (5)	0.063 (7)	0.052 (6)	−0.009 (5)	−0.002 (4)	−0.021 (5)
C6	0.045 (7)	0.087 (10)	0.061 (8)	−0.002 (7)	0.002 (6)	−0.031 (7)
C7	0.078 (9)	0.083 (9)	0.043 (6)	−0.019 (8)	−0.002 (6)	−0.019 (6)
C8	0.052 (7)	0.076 (8)	0.050 (7)	0.006 (6)	−0.017 (5)	−0.005 (6)
C9	0.033 (5)	0.064 (7)	0.054 (6)	−0.005 (5)	−0.013 (5)	−0.013 (5)
C10	0.042 (6)	0.059 (7)	0.055 (6)	0.005 (5)	−0.012 (5)	−0.019 (5)
C11	0.131 (17)	0.143 (18)	0.049 (8)	0.026 (14)	0.006 (9)	−0.009 (10)
C12	0.054 (7)	0.060 (7)	0.075 (8)	0.006 (6)	−0.019 (6)	−0.032 (6)
C13	0.029 (5)	0.031 (5)	0.045 (5)	−0.007 (4)	0.001 (4)	0.001 (4)
C14	0.031 (5)	0.022 (4)	0.051 (6)	−0.009 (4)	0.007 (4)	0.008 (4)
C15	0.031 (5)	0.039 (5)	0.058 (6)	0.003 (4)	0.001 (5)	0.014 (5)
C16	0.039 (6)	0.033 (5)	0.054 (6)	−0.013 (4)	−0.009 (5)	0.009 (4)
C17	0.037 (5)	0.032 (5)	0.044 (5)	−0.008 (4)	−0.001 (4)	−0.002 (4)
C18	0.029 (5)	0.023 (4)	0.045 (5)	−0.009 (4)	0.004 (4)	0.006 (4)
C19	0.035 (5)	0.042 (5)	0.043 (5)	0.001 (4)	−0.002 (4)	−0.001 (4)
C20	0.064 (8)	0.056 (7)	0.062 (7)	0.001 (6)	−0.021 (6)	0.000 (6)
C21	0.043 (6)	0.026 (5)	0.067 (7)	−0.005 (4)	0.002 (5)	0.002 (5)
C22	0.026 (4)	0.020 (4)	0.052 (5)	0.000 (3)	−0.001 (4)	−0.007 (4)
C23	0.050 (7)	0.032 (5)	0.077 (8)	−0.002 (5)	−0.002 (6)	−0.011 (5)
C24	0.053 (7)	0.039 (6)	0.069 (8)	0.013 (5)	0.000 (6)	−0.012 (5)
C25	0.066 (8)	0.046 (6)	0.054 (7)	−0.001 (6)	0.009 (6)	−0.017 (5)
C26	0.056 (7)	0.043 (6)	0.064 (7)	−0.010 (5)	0.017 (6)	−0.014 (5)
C27	0.106 (14)	0.057 (9)	0.098 (12)	−0.014 (9)	0.057 (11)	−0.016 (8)
C28	0.117 (15)	0.070 (10)	0.067 (10)	0.006 (10)	0.019 (10)	−0.011 (8)
C29	0.108 (13)	0.066 (9)	0.059 (8)	0.012 (9)	−0.007 (8)	−0.020 (7)
C30	0.070 (8)	0.053 (7)	0.053 (7)	0.005 (6)	−0.011 (6)	−0.012 (6)
C31	0.063 (9)	0.068 (9)	0.085 (10)	−0.002 (7)	−0.019 (7)	−0.013 (7)
C32	0.25 (3)	0.088 (14)	0.083 (13)	0.019 (17)	0.048 (17)	0.022 (11)
C33	0.053 (8)	0.087 (11)	0.105 (12)	−0.025 (8)	0.014 (8)	−0.022 (9)
C34	0.031 (5)	0.027 (5)	0.064 (7)	−0.003 (4)	0.003 (5)	0.005 (4)
C35	0.032 (5)	0.029 (5)	0.070 (7)	−0.001 (4)	−0.005 (5)	0.011 (5)
C36	0.040 (6)	0.034 (5)	0.076 (8)	−0.007 (4)	−0.023 (5)	0.009 (5)
C37	0.054 (7)	0.027 (5)	0.065 (7)	0.004 (5)	0.000 (6)	0.005 (5)
C38	0.039 (6)	0.027 (5)	0.067 (7)	0.000 (4)	0.004 (5)	0.006 (5)
C39	0.037 (5)	0.019 (4)	0.065 (7)	−0.004 (4)	−0.002 (5)	0.002 (4)
C40	0.041 (6)	0.042 (6)	0.068 (7)	−0.005 (5)	0.001 (5)	−0.007 (5)
C41	0.061 (8)	0.047 (7)	0.071 (8)	−0.007 (6)	−0.009 (6)	0.001 (6)
C42	0.033 (6)	0.053 (7)	0.085 (9)	−0.006 (5)	−0.021 (6)	0.016 (6)
C43	0.029 (5)	0.023 (4)	0.036 (5)	0.010 (3)	−0.001 (4)	0.003 (3)
C44	0.023 (4)	0.024 (4)	0.057 (6)	−0.011 (3)	−0.006 (4)	0.004 (4)
C45	0.028 (5)	0.018 (4)	0.049 (5)	−0.011 (3)	−0.004 (4)	0.004 (4)
C46	0.025 (4)	0.024 (4)	0.041 (5)	−0.001 (3)	−0.009 (4)	−0.003 (4)
C47	0.016 (4)	0.033 (5)	0.043 (5)	−0.005 (3)	−0.010 (4)	−0.007 (4)
C48	0.025 (4)	0.033 (5)	0.040 (5)	0.000 (4)	−0.010 (4)	−0.006 (4)
C49	0.034 (5)	0.036 (5)	0.040 (5)	−0.009 (4)	−0.011 (4)	−0.008 (4)
C50	0.024 (4)	0.043 (5)	0.043 (5)	−0.006 (4)	−0.010 (4)	−0.011 (4)
C51	0.030 (5)	0.037 (5)	0.050 (6)	0.000 (4)	−0.012 (4)	−0.018 (4)
C52	0.043 (6)	0.034 (5)	0.056 (6)	0.011 (4)	−0.020 (5)	−0.014 (4)
C53	0.036 (5)	0.050 (6)	0.043 (5)	−0.007 (5)	−0.008 (4)	−0.006 (5)
C54	0.021 (4)	0.026 (4)	0.049 (5)	−0.002 (3)	0.001 (4)	−0.003 (4)
C55	0.032 (5)	0.037 (5)	0.044 (5)	−0.011 (4)	−0.006 (4)	0.013 (4)
C56	0.032 (5)	0.059 (7)	0.061 (7)	−0.021 (5)	−0.016 (5)	0.035 (6)
C57	0.046 (7)	0.090 (10)	0.063 (8)	−0.013 (7)	−0.010 (6)	0.041 (8)
C58	0.052 (8)	0.138 (15)	0.040 (7)	−0.034 (9)	−0.020 (6)	0.039 (8)
C59	0.050 (7)	0.098 (10)	0.035 (6)	−0.027 (7)	0.008 (5)	0.005 (6)
C60	0.040 (6)	0.053 (6)	0.034 (5)	−0.017 (5)	0.002 (4)	0.009 (4)
C61	0.043 (6)	0.044 (6)	0.050 (6)	−0.011 (5)	0.012 (5)	−0.005 (5)
C62	0.059 (9)	0.22 (2)	0.055 (8)	−0.038 (12)	−0.027 (7)	0.021 (11)
C63	0.036 (6)	0.047 (7)	0.099 (10)	−0.002 (5)	−0.013 (6)	0.020 (7)
C64	0.053 (7)	0.084 (9)	0.042 (6)	0.020 (6)	−0.013 (5)	0.009 (6)
C65	0.060 (8)	0.097 (10)	0.039 (6)	0.021 (7)	−0.009 (5)	−0.009 (6)
C66	0.074 (10)	0.139 (16)	0.061 (9)	0.037 (10)	−0.015 (8)	0.004 (9)
C67	0.054 (8)	0.103 (11)	0.054 (7)	0.040 (8)	−0.012 (6)	−0.001 (7)
C68	0.023 (4)	0.027 (4)	0.045 (5)	−0.001 (3)	−0.006 (4)	−0.011 (4)
C69	0.027 (5)	0.036 (5)	0.038 (5)	0.001 (4)	−0.003 (4)	−0.001 (4)
Cl1	0.0457 (15)	0.0331 (13)	0.103 (2)	0.0093 (11)	−0.0084 (15)	−0.0083 (14)
Cl2	0.0299 (12)	0.0510 (14)	0.0631 (15)	0.0118 (10)	−0.0121 (11)	−0.0165 (12)
Cl3	0.0448 (13)	0.0498 (13)	0.0340 (11)	0.0043 (11)	−0.0024 (10)	−0.0036 (10)
Cl4	0.0428 (13)	0.0393 (12)	0.0382 (11)	−0.0069 (10)	−0.0108 (10)	0.0057 (9)
Cu1	0.0374 (8)	0.0315 (7)	0.0466 (8)	−0.0005 (6)	−0.0099 (6)	0.0023 (6)
Cu2	0.0360 (8)	0.0357 (7)	0.0435 (8)	0.0001 (6)	−0.0019 (6)	−0.0035 (6)
Cu3	0.0242 (6)	0.0239 (6)	0.0438 (7)	−0.0021 (5)	−0.0041 (5)	0.0003 (5)
Cu4	0.0266 (7)	0.0257 (6)	0.0412 (7)	−0.0034 (5)	−0.0022 (5)	−0.0006 (5)
Cu5	0.0272 (7)	0.0308 (6)	0.0331 (6)	−0.0008 (5)	−0.0034 (5)	−0.0013 (5)
Cu6	0.0266 (6)	0.0272 (6)	0.0342 (7)	−0.0027 (5)	−0.0050 (5)	0.0023 (5)
Cu7	0.0252 (6)	0.0321 (7)	0.0373 (7)	−0.0009 (5)	−0.0091 (5)	0.0016 (5)
Cu8	0.0305 (7)	0.0272 (6)	0.0496 (8)	0.0024 (5)	−0.0066 (6)	−0.0012 (6)
Cu9	0.0239 (7)	0.0364 (7)	0.0456 (8)	0.0035 (5)	−0.0055 (6)	−0.0057 (6)
Cu10	0.0238 (6)	0.0284 (6)	0.0344 (7)	−0.0016 (5)	−0.0009 (5)	−0.0035 (5)
Ir1	0.0281 (2)	0.02377 (19)	0.0499 (2)	−0.00047 (15)	−0.00544 (17)	−0.00182 (16)
Ir2	0.02062 (18)	0.02220 (17)	0.03170 (19)	−0.00222 (13)	−0.00376 (14)	0.00161 (13)
Ir3	0.02007 (18)	0.02605 (18)	0.03278 (19)	−0.00277 (14)	−0.00277 (14)	−0.00204 (14)
N1	0.030 (4)	0.038 (4)	0.050 (5)	−0.012 (3)	0.003 (4)	−0.007 (4)
N2	0.025 (4)	0.035 (4)	0.047 (5)	−0.007 (3)	0.002 (3)	−0.005 (3)
N3	0.036 (5)	0.056 (5)	0.039 (5)	0.004 (4)	−0.013 (4)	0.002 (4)
N4	0.064 (7)	0.137 (12)	0.046 (6)	0.028 (8)	−0.015 (5)	−0.001 (7)
N5	0.043 (5)	0.035 (4)	0.057 (5)	−0.002 (4)	−0.003 (4)	−0.008 (4)
N6	0.040 (5)	0.027 (4)	0.057 (5)	−0.001 (3)	−0.001 (4)	−0.004 (4)
N7	0.033 (4)	0.030 (4)	0.037 (4)	−0.005 (3)	−0.002 (3)	0.003 (3)
N8	0.015 (3)	0.023 (3)	0.047 (4)	−0.004 (3)	−0.007 (3)	0.003 (3)
N9	0.025 (4)	0.026 (4)	0.041 (4)	−0.003 (3)	0.000 (3)	−0.007 (3)
O1	0.110 (9)	0.093 (8)	0.066 (6)	0.013 (7)	0.012 (6)	−0.001 (6)
C71	0.086 (18)	0.075 (16)	0.061 (14)	0.032 (14)	0.015 (13)	0.008 (12)
O2	0.109 (16)	0.070 (11)	0.063 (10)	−0.002 (10)	−0.026 (10)	−0.008 (8)
C70	0.19 (4)	0.18 (4)	0.075 (17)	−0.07 (3)	−0.04 (2)	0.01 (2)
C72	0.19 (4)	0.18 (4)	0.075 (17)	−0.07 (3)	−0.04 (2)	0.01 (2)

### Ben Tickner BJT004 Geometric parameters (Å, º)

**Table:**

C1—Ir3	2.032 (9)	C47—C48	1.382 (13)
C1—N1	1.345 (13)	C47—C54	1.495 (12)
C1—N2	1.381 (12)	C48—H48	0.9500
C2—H2	0.9500	C48—C49	1.382 (13)
C2—C3	1.311 (16)	C49—C50	1.387 (14)
C2—N2	1.414 (13)	C49—C53	1.506 (14)
C3—H3	0.9500	C50—H50	0.9500
C3—N1	1.391 (13)	C50—C51	1.382 (14)
C4—C5	1.396 (15)	C51—C52	1.509 (13)
C4—C9	1.371 (17)	C52—H52A	0.9800
C4—N1	1.439 (14)	C52—H52B	0.9800
C5—C6	1.386 (18)	C52—H52C	0.9800
C5—C12	1.484 (17)	C53—H53A	0.9800
C6—H6	0.9500	C53—H53B	0.9800
C6—C7	1.39 (2)	C53—H53C	0.9800
C7—C8	1.369 (19)	C54—H54A	0.9800
C7—C11	1.531 (19)	C54—H54B	0.9800
C8—H8	0.9500	C54—H54C	0.9800
C8—C9	1.379 (17)	C55—C56	1.416 (15)
C9—C10	1.529 (15)	C55—C60	1.399 (16)
C10—H10A	0.9800	C55—N7	1.424 (13)
C10—H10B	0.9800	C56—C57	1.363 (19)
C10—H10C	0.9800	C56—C63	1.523 (19)
C11—H11A	0.9800	C57—H57	0.9500
C11—H11B	0.9800	C57—C58	1.37 (2)
C11—H11C	0.9800	C58—C59	1.38 (2)
C12—H12A	0.9800	C58—C62	1.523 (18)
C12—H12B	0.9800	C59—H59	0.9500
C12—H12C	0.9800	C59—C60	1.377 (16)
C13—C14	1.395 (14)	C60—C61	1.491 (16)
C13—C18	1.389 (14)	C61—H61A	0.9800
C13—N2	1.418 (13)	C61—H61B	0.9800
C14—C15	1.374 (16)	C61—H61C	0.9800
C14—C21	1.509 (14)	C62—H62A	0.9800
C15—H15	0.9500	C62—H62B	0.9800
C15—C16	1.377 (16)	C62—H62C	0.9800
C16—C17	1.371 (14)	C63—H63A	0.9800
C16—C20	1.524 (16)	C63—H63B	0.9800
C17—H17	0.9500	C63—H63C	0.9800
C17—C18	1.414 (14)	C64—H64	0.9500
C18—C19	1.500 (13)	C64—C65	1.366 (16)
C19—H19A	0.9800	C64—N3	1.316 (15)
C19—H19B	0.9800	C65—H65	0.9500
C19—H19C	0.9800	C65—N4	1.309 (17)
C20—H20A	0.9800	C66—H66	0.9500
C20—H20B	0.9800	C66—C67	1.350 (19)
C20—H20C	0.9800	C66—N4	1.328 (19)
C21—H21A	0.9800	C67—H67	0.9500
C21—H21B	0.9800	C67—N3	1.370 (15)
C21—H21C	0.9800	C68—H68	0.9500
C22—Ir1	2.054 (8)	C68—C69	1.384 (13)
C22—N5	1.350 (13)	C68—N9	1.337 (12)
C22—N6	1.366 (12)	C69—H69	0.9500
C23—H23	0.9500	C69—N9^i^	1.345 (12)
C23—C24	1.338 (18)	Cl1—Cu8	2.127 (3)
C23—N6	1.377 (14)	Cl2—Cu9	2.129 (3)
C24—H24	0.9500	Cl3—Cu2	2.183 (3)
C24—N5	1.413 (14)	Cl3—Cu5	2.290 (3)
C25—C26	1.370 (18)	Cl4—Cu1	2.172 (3)
C25—C30	1.429 (19)	Cl4—Cu6	2.287 (3)
C25—N5	1.449 (15)	Cu1—Cu3	2.8138 (19)
C26—C27	1.39 (2)	Cu1—Cu4	2.716 (2)
C26—C33	1.49 (2)	Cu1—Cu6	2.6274 (18)
C27—H27	0.9500	Cu1—Ir1	2.5227 (15)
C27—C28	1.41 (3)	Cu2—Cu3	2.716 (2)
C28—C29	1.36 (3)	Cu2—Cu4	2.8052 (19)
C28—C32	1.53 (2)	Cu2—Cu5	2.5877 (18)
C29—H29	0.9500	Cu2—Ir1	2.5338 (15)
C29—C30	1.39 (2)	Cu3—Cu4	2.4916 (18)
C30—C31	1.49 (2)	Cu3—Cu5	2.6364 (18)
C31—H31A	0.9800	Cu3—Cu6	2.6145 (18)
C31—H31B	0.9800	Cu3—Ir1	2.6325 (13)
C31—H31C	0.9800	Cu3—Ir2	2.5665 (13)
C32—H32A	0.9800	Cu4—Cu5	2.5860 (18)
C32—H32B	0.9800	Cu4—Cu6	2.6325 (18)
C32—H32C	0.9800	Cu4—Ir1	2.7478 (13)
C33—H33A	0.9800	Cu4—Ir3	2.6337 (13)
C33—H33B	0.9800	Cu5—Cu7	2.5597 (18)
C33—H33C	0.9800	Cu5—Ir2	2.6856 (13)
C34—C35	1.393 (16)	Cu5—Ir3	2.6585 (13)
C34—C39	1.413 (14)	Cu6—Cu10	2.5899 (17)
C34—N6	1.440 (14)	Cu6—Ir2	2.6763 (12)
C35—C36	1.384 (17)	Cu6—Ir3	2.6559 (13)
C35—C42	1.513 (15)	Cu7—Cu8	2.5971 (19)
C36—H36	0.9500	Cu7—Cu9	2.5615 (19)
C36—C37	1.387 (16)	Cu7—Cu10	3.0417 (18)
C37—C38	1.400 (17)	Cu7—Ir2	2.6089 (13)
C37—C41	1.499 (18)	Cu7—Ir3	2.6462 (13)
C38—H38	0.9500	Cu7—N3	1.992 (8)
C38—C39	1.368 (16)	Cu8—Cu10	2.6155 (18)
C39—C40	1.517 (16)	Cu8—Ir2	2.5304 (13)
C40—H40A	0.9800	Cu9—Cu10	2.5802 (18)
C40—H40B	0.9800	Cu9—Ir3	2.5350 (13)
C40—H40C	0.9800	Cu10—Ir2	2.6378 (13)
C41—H41A	0.9800	Cu10—Ir3	2.6847 (13)
C41—H41B	0.9800	Cu10—N9	2.014 (7)
C41—H41C	0.9800	O1—H1A	0.8400
C42—H42A	0.9800	O1—H1B	0.8400
C42—H42B	0.9800	O1—C70	1.45 (3)
C42—H42C	0.9800	O1—C72	1.48 (5)
C43—Ir2	2.022 (9)	C71—H71A	0.9800
C43—N7	1.372 (11)	C71—H71B	0.9800
C43—N8	1.363 (12)	C71—H71C	0.9800
C44—H44	0.9500	C71—O2	1.40 (3)
C44—C45	1.340 (14)	O2—H2A	0.8400
C44—N7	1.408 (12)	C70—H70A	0.9800
C45—H45	0.9500	C70—H70B	0.9800
C45—N8	1.412 (11)	C70—H70C	0.9800
C46—C47	1.401 (12)	C72—H72A	0.9800
C46—C51	1.389 (13)	C72—H72B	0.9800
C46—N8	1.446 (12)	C72—H72C	0.9800
			
N1—C1—Ir3	129.6 (7)	N9^i^—C69—H69	119.3
N1—C1—N2	105.5 (8)	Cu2—Cl3—Cu5	70.64 (8)
N2—C1—Ir3	124.9 (7)	Cu1—Cl4—Cu6	72.15 (8)
C3—C2—H2	126.2	Cl4—Cu1—Cu3	106.82 (8)
C3—C2—N2	107.6 (9)	Cl4—Cu1—Cu4	108.26 (9)
N2—C2—H2	126.2	Cl4—Cu1—Cu6	55.94 (7)
C2—C3—H3	126.0	Cl4—Cu1—Ir1	165.57 (9)
C2—C3—N1	108.1 (10)	Cu4—Cu1—Cu3	53.52 (5)
N1—C3—H3	126.0	Cu6—Cu1—Cu3	57.31 (5)
C5—C4—N1	117.9 (10)	Cu6—Cu1—Cu4	59.00 (5)
C9—C4—C5	123.0 (11)	Ir1—Cu1—Cu3	58.81 (4)
C9—C4—N1	119.1 (9)	Ir1—Cu1—Cu4	63.14 (4)
C4—C5—C12	122.8 (11)	Ir1—Cu1—Cu6	110.59 (6)
C6—C5—C4	115.9 (11)	Cl3—Cu2—Cu3	111.84 (9)
C6—C5—C12	121.3 (11)	Cl3—Cu2—Cu4	104.44 (9)
C5—C6—H6	118.6	Cl3—Cu2—Cu5	56.62 (7)
C7—C6—C5	122.7 (11)	Cl3—Cu2—Ir1	166.10 (9)
C7—C6—H6	118.6	Cu3—Cu2—Cu4	53.62 (5)
C6—C7—C11	120.9 (13)	Cu5—Cu2—Cu3	59.56 (5)
C8—C7—C6	118.3 (12)	Cu5—Cu2—Cu4	57.14 (5)
C8—C7—C11	120.7 (15)	Ir1—Cu2—Cu3	60.07 (4)
C7—C8—H8	119.2	Ir1—Cu2—Cu4	61.70 (4)
C7—C8—C9	121.5 (12)	Ir1—Cu2—Cu5	110.96 (6)
C9—C8—H8	119.2	Cu2—Cu3—Cu1	104.92 (6)
C4—C9—C8	118.4 (11)	Cu4—Cu3—Cu1	61.23 (5)
C4—C9—C10	121.2 (10)	Cu4—Cu3—Cu2	65.02 (5)
C8—C9—C10	120.4 (11)	Cu4—Cu3—Cu5	60.49 (5)
C9—C10—H10A	109.5	Cu4—Cu3—Cu6	62.01 (5)
C9—C10—H10B	109.5	Cu4—Cu3—Ir1	64.79 (4)
C9—C10—H10C	109.5	Cu4—Cu3—Ir2	99.09 (5)
H10A—C10—H10B	109.5	Cu5—Cu3—Cu1	120.76 (6)
H10A—C10—H10C	109.5	Cu5—Cu3—Cu2	57.80 (5)
H10B—C10—H10C	109.5	Cu6—Cu3—Cu1	57.76 (5)
C7—C11—H11A	109.5	Cu6—Cu3—Cu2	125.79 (6)
C7—C11—H11B	109.5	Cu6—Cu3—Cu5	86.14 (5)
C7—C11—H11C	109.5	Cu6—Cu3—Ir1	107.61 (5)
H11A—C11—H11B	109.5	Ir1—Cu3—Cu1	55.07 (4)
H11A—C11—H11C	109.5	Ir1—Cu3—Cu2	56.53 (4)
H11B—C11—H11C	109.5	Ir1—Cu3—Cu5	106.43 (5)
C5—C12—H12A	109.5	Ir2—Cu3—Cu1	119.07 (6)
C5—C12—H12B	109.5	Ir2—Cu3—Cu2	117.60 (6)
C5—C12—H12C	109.5	Ir2—Cu3—Cu5	62.14 (4)
H12A—C12—H12B	109.5	Ir2—Cu3—Cu6	62.20 (4)
H12A—C12—H12C	109.5	Ir2—Cu3—Ir1	163.87 (6)
H12B—C12—H12C	109.5	Cu1—Cu4—Cu2	105.15 (6)
C14—C13—N2	117.8 (9)	Cu1—Cu4—Ir1	54.99 (4)
C18—C13—C14	122.4 (10)	Cu3—Cu4—Cu1	65.24 (5)
C18—C13—N2	119.7 (9)	Cu3—Cu4—Cu2	61.36 (5)
C13—C14—C21	122.6 (10)	Cu3—Cu4—Cu5	62.53 (5)
C15—C14—C13	117.7 (9)	Cu3—Cu4—Cu6	61.29 (5)
C15—C14—C21	119.6 (9)	Cu3—Cu4—Ir1	60.09 (4)
C14—C15—H15	118.7	Cu3—Cu4—Ir3	97.57 (5)
C14—C15—C16	122.5 (9)	Cu5—Cu4—Cu1	126.68 (6)
C16—C15—H15	118.7	Cu5—Cu4—Cu2	57.19 (5)
C15—C16—C20	120.5 (10)	Cu5—Cu4—Cu6	86.80 (5)
C17—C16—C15	118.7 (10)	Cu5—Cu4—Ir1	104.55 (5)
C17—C16—C20	120.8 (10)	Cu5—Cu4—Ir3	61.23 (4)
C16—C17—H17	119.0	Cu6—Cu4—Cu1	58.82 (5)
C16—C17—C18	121.9 (9)	Cu6—Cu4—Cu2	121.51 (6)
C18—C17—H17	119.0	Cu6—Cu4—Ir1	103.79 (5)
C13—C18—C17	116.7 (9)	Cu6—Cu4—Ir3	60.57 (4)
C13—C18—C19	123.4 (9)	Ir1—Cu4—Cu2	54.28 (4)
C17—C18—C19	119.8 (9)	Ir3—Cu4—Cu1	117.58 (6)
C18—C19—H19A	109.5	Ir3—Cu4—Cu2	117.81 (5)
C18—C19—H19B	109.5	Ir3—Cu4—Ir1	157.65 (5)
C18—C19—H19C	109.5	Cl3—Cu5—Cu2	52.74 (7)
H19A—C19—H19B	109.5	Cl3—Cu5—Cu3	111.15 (8)
H19A—C19—H19C	109.5	Cl3—Cu5—Cu4	108.49 (8)
H19B—C19—H19C	109.5	Cl3—Cu5—Cu7	124.87 (9)
C16—C20—H20A	109.5	Cl3—Cu5—Ir2	143.50 (9)
C16—C20—H20B	109.5	Cl3—Cu5—Ir3	141.38 (9)
C16—C20—H20C	109.5	Cu2—Cu5—Cu3	62.64 (5)
H20A—C20—H20B	109.5	Cu2—Cu5—Ir2	117.95 (6)
H20A—C20—H20C	109.5	Cu2—Cu5—Ir3	125.23 (6)
H20B—C20—H20C	109.5	Cu3—Cu5—Ir2	57.65 (4)
C14—C21—H21A	109.5	Cu3—Cu5—Ir3	93.50 (5)
C14—C21—H21B	109.5	Cu4—Cu5—Cu2	65.67 (5)
C14—C21—H21C	109.5	Cu4—Cu5—Cu3	56.98 (5)
H21A—C21—H21B	109.5	Cu4—Cu5—Ir2	93.78 (5)
H21A—C21—H21C	109.5	Cu4—Cu5—Ir3	60.27 (4)
H21B—C21—H21C	109.5	Cu7—Cu5—Cu2	173.38 (7)
N5—C22—Ir1	127.8 (7)	Cu7—Cu5—Cu3	116.42 (6)
N5—C22—N6	104.1 (8)	Cu7—Cu5—Cu4	119.86 (6)
N6—C22—Ir1	127.8 (7)	Cu7—Cu5—Ir2	59.59 (4)
C24—C23—H23	126.2	Cu7—Cu5—Ir3	60.91 (4)
C24—C23—N6	107.5 (10)	Ir3—Cu5—Ir2	74.79 (3)
N6—C23—H23	126.2	Cl4—Cu6—Cu1	51.90 (7)
C23—C24—H24	127.0	Cl4—Cu6—Cu3	110.10 (8)
C23—C24—N5	105.9 (10)	Cl4—Cu6—Cu4	107.57 (8)
N5—C24—H24	127.0	Cl4—Cu6—Cu10	125.49 (9)
C26—C25—C30	123.6 (12)	Cl4—Cu6—Ir2	144.79 (8)
C26—C25—N5	120.1 (12)	Cl4—Cu6—Ir3	140.09 (8)
C30—C25—N5	116.3 (11)	Cu1—Cu6—Cu4	62.18 (5)
C25—C26—C27	116.1 (15)	Cu1—Cu6—Ir2	122.02 (6)
C25—C26—C33	121.4 (12)	Cu1—Cu6—Ir3	120.01 (6)
C27—C26—C33	122.5 (14)	Cu3—Cu6—Cu1	64.93 (5)
C26—C27—H27	118.5	Cu3—Cu6—Cu4	56.70 (5)
C26—C27—C28	123.0 (16)	Cu3—Cu6—Ir2	58.02 (4)
C28—C27—H27	118.5	Cu3—Cu6—Ir3	94.07 (5)
C27—C28—C32	120 (2)	Cu4—Cu6—Ir2	92.94 (5)
C29—C28—C27	118.2 (16)	Cu4—Cu6—Ir3	59.74 (4)
C29—C28—C32	122 (2)	Cu10—Cu6—Cu1	177.38 (7)
C28—C29—H29	118.8	Cu10—Cu6—Cu3	117.42 (6)
C28—C29—C30	122.4 (17)	Cu10—Cu6—Cu4	119.86 (6)
C30—C29—H29	118.8	Cu10—Cu6—Ir2	60.09 (4)
C25—C30—C31	122.4 (12)	Cu10—Cu6—Ir3	61.55 (4)
C29—C30—C25	116.5 (14)	Ir3—Cu6—Ir2	74.99 (3)
C29—C30—C31	121.0 (14)	Cu5—Cu7—Cu8	120.17 (6)
C30—C31—H31A	109.5	Cu5—Cu7—Cu9	119.11 (6)
C30—C31—H31B	109.5	Cu5—Cu7—Cu10	97.15 (5)
C30—C31—H31C	109.5	Cu5—Cu7—Ir2	62.60 (4)
H31A—C31—H31B	109.5	Cu5—Cu7—Ir3	61.39 (4)
H31A—C31—H31C	109.5	Cu8—Cu7—Cu10	54.58 (5)
H31B—C31—H31C	109.5	Cu8—Cu7—Ir2	58.16 (4)
C28—C32—H32A	109.5	Cu8—Cu7—Ir3	109.79 (6)
C28—C32—H32B	109.5	Cu9—Cu7—Cu8	87.55 (6)
C28—C32—H32C	109.5	Cu9—Cu7—Cu10	54.01 (5)
H32A—C32—H32B	109.5	Cu9—Cu7—Ir2	108.44 (6)
H32A—C32—H32C	109.5	Cu9—Cu7—Ir3	58.23 (4)
H32B—C32—H32C	109.5	Ir2—Cu7—Cu10	55.01 (3)
C26—C33—H33A	109.5	Ir2—Cu7—Ir3	76.28 (3)
C26—C33—H33B	109.5	Ir3—Cu7—Cu10	55.81 (3)
C26—C33—H33C	109.5	N3—Cu7—Cu5	111.3 (3)
H33A—C33—H33B	109.5	N3—Cu7—Cu8	112.0 (3)
H33A—C33—H33C	109.5	N3—Cu7—Cu9	103.8 (3)
H33B—C33—H33C	109.5	N3—Cu7—Cu10	150.8 (3)
C35—C34—C39	122.5 (10)	N3—Cu7—Ir2	145.4 (3)
C35—C34—N6	118.9 (9)	N3—Cu7—Ir3	133.3 (3)
C39—C34—N6	118.7 (10)	Cl1—Cu8—Cu7	127.85 (11)
C34—C35—C42	119.9 (11)	Cl1—Cu8—Cu10	122.14 (11)
C36—C35—C34	118.0 (10)	Cl1—Cu8—Ir2	170.41 (11)
C36—C35—C42	122.1 (11)	Cu7—Cu8—Cu10	71.40 (5)
C35—C36—H36	119.2	Ir2—Cu8—Cu7	61.15 (4)
C35—C36—C37	121.6 (11)	Ir2—Cu8—Cu10	61.65 (4)
C37—C36—H36	119.2	Cl2—Cu9—Cu7	118.91 (10)
C36—C37—C38	118.1 (11)	Cl2—Cu9—Cu10	118.20 (10)
C36—C37—C41	120.2 (12)	Cl2—Cu9—Ir3	177.99 (10)
C38—C37—C41	121.6 (11)	Cu7—Cu9—Cu10	72.54 (5)
C37—C38—H38	118.5	Ir3—Cu9—Cu7	62.56 (4)
C39—C38—C37	123.1 (10)	Ir3—Cu9—Cu10	63.31 (4)
C39—C38—H38	118.5	Cu6—Cu10—Cu7	94.99 (5)
C34—C39—C40	120.7 (10)	Cu6—Cu10—Cu8	118.77 (6)
C38—C39—C34	116.5 (10)	Cu6—Cu10—Ir2	61.58 (4)
C38—C39—C40	122.8 (10)	Cu6—Cu10—Ir3	60.43 (4)
C39—C40—H40A	109.5	Cu8—Cu10—Cu7	54.02 (5)
C39—C40—H40B	109.5	Cu8—Cu10—Ir2	57.59 (4)
C39—C40—H40C	109.5	Cu8—Cu10—Ir3	108.06 (5)
H40A—C40—H40B	109.5	Cu9—Cu10—Cu6	117.58 (6)
H40A—C40—H40C	109.5	Cu9—Cu10—Cu7	53.45 (5)
H40B—C40—H40C	109.5	Cu9—Cu10—Cu8	86.77 (6)
C37—C41—H41A	109.5	Cu9—Cu10—Ir2	107.00 (5)
C37—C41—H41B	109.5	Cu9—Cu10—Ir3	57.52 (4)
C37—C41—H41C	109.5	Ir2—Cu10—Cu7	54.13 (3)
H41A—C41—H41B	109.5	Ir2—Cu10—Ir3	75.14 (3)
H41A—C41—H41C	109.5	Ir3—Cu10—Cu7	54.62 (3)
H41B—C41—H41C	109.5	N9—Cu10—Cu6	123.1 (2)
C35—C42—H42A	109.5	N9—Cu10—Cu7	141.8 (2)
C35—C42—H42B	109.5	N9—Cu10—Cu8	102.9 (2)
C35—C42—H42C	109.5	N9—Cu10—Cu9	100.7 (2)
H42A—C42—H42B	109.5	N9—Cu10—Ir2	144.2 (2)
H42A—C42—H42C	109.5	N9—Cu10—Ir3	140.1 (2)
H42B—C42—H42C	109.5	C22—Ir1—Cu1	122.8 (3)
N7—C43—Ir2	127.5 (7)	C22—Ir1—Cu2	101.9 (3)
N8—C43—Ir2	127.6 (6)	C22—Ir1—Cu3	164.6 (3)
N8—C43—N7	104.8 (8)	C22—Ir1—Cu4	115.7 (3)
C45—C44—H44	126.1	Cu1—Ir1—Cu2	120.28 (5)
C45—C44—N7	107.8 (8)	Cu1—Ir1—Cu3	66.12 (4)
N7—C44—H44	126.1	Cu1—Ir1—Cu4	61.87 (4)
C44—C45—H45	126.8	Cu2—Ir1—Cu3	63.40 (4)
C44—C45—N8	106.4 (8)	Cu2—Ir1—Cu4	64.01 (4)
N8—C45—H45	126.8	Cu3—Ir1—Cu4	55.12 (4)
C47—C46—N8	118.2 (8)	C43—Ir2—Cu3	92.8 (2)
C51—C46—C47	122.2 (9)	C43—Ir2—Cu5	124.8 (3)
C51—C46—N8	119.6 (8)	C43—Ir2—Cu6	125.2 (2)
C46—C47—C54	121.2 (8)	C43—Ir2—Cu7	130.7 (2)
C48—C47—C46	116.9 (8)	C43—Ir2—Cu8	89.9 (2)
C48—C47—C54	121.9 (8)	C43—Ir2—Cu10	130.5 (3)
C47—C48—H48	118.5	Cu3—Ir2—Cu5	60.21 (4)
C49—C48—C47	122.9 (9)	Cu3—Ir2—Cu6	59.78 (4)
C49—C48—H48	118.5	Cu3—Ir2—Cu7	117.17 (4)
C48—C49—C50	117.7 (9)	Cu3—Ir2—Cu10	117.42 (4)
C48—C49—C53	120.7 (9)	Cu6—Ir2—Cu5	83.94 (4)
C50—C49—C53	121.7 (9)	Cu7—Ir2—Cu5	57.80 (4)
C49—C50—H50	118.8	Cu7—Ir2—Cu6	103.93 (4)
C51—C50—C49	122.4 (9)	Cu7—Ir2—Cu10	70.86 (4)
C51—C50—H50	118.8	Cu8—Ir2—Cu3	177.35 (4)
C46—C51—C52	121.9 (9)	Cu8—Ir2—Cu5	117.92 (4)
C50—C51—C46	117.6 (9)	Cu8—Ir2—Cu6	118.69 (4)
C50—C51—C52	120.5 (9)	Cu8—Ir2—Cu7	60.68 (4)
C51—C52—H52A	109.5	Cu8—Ir2—Cu10	60.76 (4)
C51—C52—H52B	109.5	Cu10—Ir2—Cu5	104.60 (4)
C51—C52—H52C	109.5	Cu10—Ir2—Cu6	58.33 (4)
H52A—C52—H52B	109.5	C1—Ir3—Cu4	98.4 (3)
H52A—C52—H52C	109.5	C1—Ir3—Cu5	126.9 (3)
H52B—C52—H52C	109.5	C1—Ir3—Cu6	126.7 (3)
C49—C53—H53A	109.5	C1—Ir3—Cu7	129.2 (3)
C49—C53—H53B	109.5	C1—Ir3—Cu9	88.4 (3)
C49—C53—H53C	109.5	C1—Ir3—Cu10	128.4 (3)
H53A—C53—H53B	109.5	Cu4—Ir3—Cu5	58.50 (4)
H53A—C53—H53C	109.5	Cu4—Ir3—Cu6	59.69 (4)
H53B—C53—H53C	109.5	Cu4—Ir3—Cu7	115.00 (4)
C47—C54—H54A	109.5	Cu4—Ir3—Cu10	116.38 (4)
C47—C54—H54B	109.5	Cu5—Ir3—Cu10	104.05 (4)
C47—C54—H54C	109.5	Cu6—Ir3—Cu5	84.87 (4)
H54A—C54—H54B	109.5	Cu6—Ir3—Cu10	58.02 (4)
H54A—C54—H54C	109.5	Cu7—Ir3—Cu5	57.70 (4)
H54B—C54—H54C	109.5	Cu7—Ir3—Cu6	103.47 (4)
C56—C55—N7	118.5 (10)	Cu7—Ir3—Cu10	69.58 (4)
C60—C55—C56	121.5 (10)	Cu9—Ir3—Cu4	173.20 (4)
C60—C55—N7	120.0 (9)	Cu9—Ir3—Cu5	116.43 (4)
C55—C56—C63	120.5 (10)	Cu9—Ir3—Cu6	116.80 (4)
C57—C56—C55	116.8 (13)	Cu9—Ir3—Cu7	59.21 (4)
C57—C56—C63	122.6 (12)	Cu9—Ir3—Cu10	59.17 (4)
C56—C57—H57	117.9	C1—N1—C3	110.4 (9)
C56—C57—C58	124.1 (13)	C1—N1—C4	127.0 (8)
C58—C57—H57	117.9	C3—N1—C4	122.6 (9)
C57—C58—C59	117.2 (12)	C1—N2—C2	108.4 (8)
C57—C58—C62	120.7 (16)	C1—N2—C13	127.5 (8)
C59—C58—C62	122.0 (18)	C2—N2—C13	123.8 (8)
C58—C59—H59	118.4	C64—N3—C67	114.9 (10)
C60—C59—C58	123.2 (14)	C64—N3—Cu7	117.9 (7)
C60—C59—H59	118.4	C67—N3—Cu7	124.4 (8)
C55—C60—C61	120.7 (9)	C65—N4—C66	115.2 (11)
C59—C60—C55	117.2 (11)	C22—N5—C24	111.0 (9)
C59—C60—C61	122.0 (11)	C22—N5—C25	126.6 (8)
C60—C61—H61A	109.5	C24—N5—C25	122.2 (9)
C60—C61—H61B	109.5	C22—N6—C23	111.3 (9)
C60—C61—H61C	109.5	C22—N6—C34	125.3 (8)
H61A—C61—H61B	109.5	C23—N6—C34	123.2 (9)
H61A—C61—H61C	109.5	C43—N7—C44	110.1 (8)
H61B—C61—H61C	109.5	C43—N7—C55	126.4 (8)
C58—C62—H62A	109.5	C44—N7—C55	123.5 (8)
C58—C62—H62B	109.5	C43—N8—C45	111.0 (8)
C58—C62—H62C	109.5	C43—N8—C46	124.5 (7)
H62A—C62—H62B	109.5	C45—N8—C46	124.4 (7)
H62A—C62—H62C	109.5	C68—N9—C69^i^	116.6 (8)
H62B—C62—H62C	109.5	C68—N9—Cu10	120.5 (6)
C56—C63—H63A	109.5	C69^i^—N9—Cu10	122.8 (6)
C56—C63—H63B	109.5	C70—O1—H1A	109.5
C56—C63—H63C	109.5	C72—O1—H1B	109.5
H63A—C63—H63B	109.5	H71A—C71—H71B	109.5
H63A—C63—H63C	109.5	H71A—C71—H71C	109.5
H63B—C63—H63C	109.5	H71B—C71—H71C	109.5
C65—C64—H64	118.9	O2—C71—H71A	109.5
N3—C64—H64	118.9	O2—C71—H71B	109.5
N3—C64—C65	122.3 (11)	O2—C71—H71C	109.5
C64—C65—H65	118.6	C71—O2—H2A	109.5
N4—C65—C64	122.8 (12)	O1—C70—H70A	109.5
N4—C65—H65	118.6	O1—C70—H70B	109.5
C67—C66—H66	118.3	O1—C70—H70C	109.5
N4—C66—H66	118.3	H70A—C70—H70B	109.5
N4—C66—C67	123.4 (14)	H70A—C70—H70C	109.5
C66—C67—H67	119.8	H70B—C70—H70C	109.5
C66—C67—N3	120.5 (13)	O1—C72—H72A	109.5
N3—C67—H67	119.8	O1—C72—H72B	109.5
C69—C68—H68	119.1	O1—C72—H72C	109.5
N9—C68—H68	119.1	H72A—C72—H72B	109.5
N9—C68—C69	121.9 (8)	H72A—C72—H72C	109.5
C68—C69—H69	119.3	H72B—C72—H72C	109.5
N9^i^—C69—C68	121.5 (9)		
			
C2—C3—N1—C1	−1.4 (13)	C49—C50—C51—C52	179.1 (9)
C2—C3—N1—C4	179.6 (10)	C51—C46—C47—C48	−5.3 (13)
C3—C2—N2—C1	0.0 (12)	C51—C46—C47—C54	176.8 (8)
C3—C2—N2—C13	174.9 (9)	C51—C46—N8—C43	85.6 (11)
C4—C5—C6—C7	−3.0 (18)	C51—C46—N8—C45	−97.1 (10)
C5—C4—C9—C8	2.0 (17)	C53—C49—C50—C51	177.0 (9)
C5—C4—C9—C10	−177.3 (10)	C54—C47—C48—C49	177.8 (8)
C5—C4—N1—C1	−86.3 (13)	C55—C56—C57—C58	−1.8 (17)
C5—C4—N1—C3	92.5 (12)	C56—C55—C60—C59	−0.3 (14)
C5—C6—C7—C8	5 (2)	C56—C55—C60—C61	−176.9 (9)
C5—C6—C7—C11	−176.8 (15)	C56—C55—N7—C43	−89.4 (11)
C6—C7—C8—C9	−4 (2)	C56—C55—N7—C44	93.1 (11)
C7—C8—C9—C4	0.4 (19)	C56—C57—C58—C59	4 (2)
C7—C8—C9—C10	179.7 (12)	C56—C57—C58—C62	−178.1 (12)
C9—C4—C5—C6	−0.7 (16)	C57—C58—C59—C60	−3.9 (19)
C9—C4—C5—C12	176.5 (11)	C58—C59—C60—C55	2.3 (17)
C9—C4—N1—C1	95.7 (13)	C58—C59—C60—C61	178.9 (11)
C9—C4—N1—C3	−85.5 (13)	C60—C55—C56—C57	0.0 (14)
C11—C7—C8—C9	178.2 (15)	C60—C55—C56—C63	176.0 (9)
C12—C5—C6—C7	179.8 (12)	C60—C55—N7—C43	90.0 (12)
C13—C14—C15—C16	−0.3 (14)	C60—C55—N7—C44	−87.5 (11)
C14—C13—C18—C17	−1.7 (13)	C62—C58—C59—C60	177.8 (12)
C14—C13—C18—C19	−179.5 (8)	C63—C56—C57—C58	−177.7 (12)
C14—C13—N2—C1	85.1 (12)	C64—C65—N4—C66	5 (3)
C14—C13—N2—C2	−88.8 (11)	C65—C64—N3—C67	−9 (2)
C14—C15—C16—C17	−0.9 (15)	C65—C64—N3—Cu7	−170.7 (12)
C14—C15—C16—C20	176.6 (10)	C66—C67—N3—C64	11 (2)
C15—C16—C17—C18	0.9 (14)	C66—C67—N3—Cu7	171.4 (14)
C16—C17—C18—C13	0.4 (13)	C67—C66—N4—C65	−3 (3)
C16—C17—C18—C19	178.3 (9)	C69—C68—N9—C69^i^	−1.0 (14)
C18—C13—C14—C15	1.7 (13)	C69—C68—N9—Cu10	176.7 (7)
C18—C13—C14—C21	178.7 (8)	Ir1—C22—N5—C24	−173.9 (8)
C18—C13—N2—C1	−93.6 (11)	Ir1—C22—N5—C25	1.6 (16)
C18—C13—N2—C2	92.5 (11)	Ir1—C22—N6—C23	174.8 (8)
C20—C16—C17—C18	−176.6 (9)	Ir1—C22—N6—C34	−1.1 (14)
C21—C14—C15—C16	−177.4 (9)	Ir2—C43—N7—C44	175.2 (6)
C23—C24—N5—C22	−1.3 (14)	Ir2—C43—N7—C55	−2.5 (13)
C23—C24—N5—C25	−177.1 (11)	Ir2—C43—N8—C45	−175.4 (6)
C24—C23—N6—C22	−1.1 (13)	Ir2—C43—N8—C46	2.2 (12)
C24—C23—N6—C34	174.9 (10)	Ir3—C1—N1—C3	179.5 (7)
C25—C26—C27—C28	1 (2)	Ir3—C1—N1—C4	−1.6 (15)
C26—C25—C30—C29	3.0 (18)	Ir3—C1—N2—C2	−179.1 (7)
C26—C25—C30—C31	179.6 (12)	Ir3—C1—N2—C13	6.2 (13)
C26—C25—N5—C22	−85.4 (14)	N1—C1—N2—C2	−0.8 (10)
C26—C25—N5—C24	89.7 (14)	N1—C1—N2—C13	−175.5 (9)
C26—C27—C28—C29	1 (2)	N1—C4—C5—C6	−178.6 (10)
C26—C27—C28—C32	−177.0 (14)	N1—C4—C5—C12	−1.4 (15)
C27—C28—C29—C30	−2 (2)	N1—C4—C9—C8	179.8 (10)
C28—C29—C30—C25	0 (2)	N1—C4—C9—C10	0.5 (15)
C28—C29—C30—C31	−177.1 (14)	N2—C1—N1—C3	1.4 (11)
C30—C25—C26—C27	−3.3 (18)	N2—C1—N1—C4	−179.7 (9)
C30—C25—C26—C33	178.2 (12)	N2—C2—C3—N1	0.9 (13)
C30—C25—N5—C22	93.7 (13)	N2—C13—C14—C15	−177.0 (8)
C30—C25—N5—C24	−91.2 (13)	N2—C13—C14—C21	0.0 (13)
C32—C28—C29—C30	176.6 (15)	N2—C13—C18—C17	176.9 (8)
C33—C26—C27—C28	179.6 (14)	N2—C13—C18—C19	−0.9 (13)
C34—C35—C36—C37	0.1 (15)	N3—C64—C65—N4	1 (3)
C35—C34—C39—C38	−3.9 (14)	N4—C66—C67—N3	−5 (3)
C35—C34—C39—C40	176.9 (9)	N5—C22—N6—C23	0.3 (11)
C35—C34—N6—C22	86.5 (12)	N5—C22—N6—C34	−175.6 (9)
C35—C34—N6—C23	−88.9 (13)	N5—C25—C26—C27	175.8 (11)
C35—C36—C37—C38	−3.0 (15)	N5—C25—C26—C33	−2.7 (17)
C35—C36—C37—C41	176.2 (10)	N5—C25—C30—C29	−176.1 (10)
C36—C37—C38—C39	2.5 (15)	N5—C25—C30—C31	0.5 (17)
C37—C38—C39—C34	0.8 (14)	N6—C22—N5—C24	0.6 (11)
C37—C38—C39—C40	−180.0 (9)	N6—C22—N5—C25	176.2 (10)
C39—C34—C35—C36	3.4 (15)	N6—C23—C24—N5	1.4 (14)
C39—C34—C35—C42	−178.8 (9)	N6—C34—C35—C36	−176.9 (9)
C39—C34—N6—C22	−93.8 (12)	N6—C34—C35—C42	0.9 (14)
C39—C34—N6—C23	90.8 (12)	N6—C34—C39—C38	176.5 (8)
C41—C37—C38—C39	−176.6 (10)	N6—C34—C39—C40	−2.8 (13)
C42—C35—C36—C37	−177.6 (10)	N7—C43—N8—C45	1.2 (9)
C44—C45—N8—C43	−0.6 (10)	N7—C43—N8—C46	178.8 (7)
C44—C45—N8—C46	−178.2 (8)	N7—C44—C45—N8	−0.3 (10)
C45—C44—N7—C43	1.1 (10)	N7—C55—C56—C57	179.4 (9)
C45—C44—N7—C55	178.9 (9)	N7—C55—C56—C63	−4.6 (13)
C46—C47—C48—C49	−0.1 (13)	N7—C55—C60—C59	−179.7 (9)
C47—C46—C51—C50	6.6 (13)	N7—C55—C60—C61	3.7 (14)
C47—C46—C51—C52	−175.1 (8)	N8—C43—N7—C44	−1.4 (9)
C47—C46—N8—C43	−93.9 (10)	N8—C43—N7—C55	−179.2 (8)
C47—C46—N8—C45	83.4 (10)	N8—C46—C47—C48	174.2 (7)
C47—C48—C49—C50	3.8 (14)	N8—C46—C47—C54	−3.7 (12)
C47—C48—C49—C53	−175.6 (9)	N8—C46—C51—C50	−172.9 (8)
C48—C49—C50—C51	−2.4 (14)	N8—C46—C51—C52	5.4 (13)
C49—C50—C51—C46	−2.6 (14)	N9—C68—C69—N9^i^	1.0 (15)

Symmetry code: (i) −*x*+2, −*y*, −*z*+1.

### Ben Tickner BJT004 Hydrogen-bond geometry (Å, º)

**Table:**

*D*—H···*A*	*D*—H	H···*A*	*D*···*A*	*D*—H···*A*
O2—H2*A*···O1	0.84	1.87	2.62 (2)	148

## References

[bb1] Adams, R. D., Chen, M., Elpitiya, G., Yang, X. & Zhang, Q. (2013). *Organometallics*, **32**, 2416–2426.

[bb2] Baumgartner, M., Schmalle, H. & Dubler, E. (1990). *Polyhedron*, **9**, 1155–1164.

[bb3] Clark, R. C. & Reid, J. S. (1995). *Acta Cryst.* A**51**, 887–897.

[bb4] Croizat, P., Sculfort, S., Welter, R. & Braunstein, P. (2016). *Organometallics*, **35**, 3949–3958.

[bb5] Dhayal, R. S., van Zyl, W. E. & Liu, C. W. (2016). *Acc. Chem. Res.* **49**, 86–95. 10.1021/acs.accounts.5b00375 26696469

[bb6] Dolomanov, O. V., Bourhis, L. J., Gildea, R. J., Howard, J. A. K. & Puschmann, H. (2009). *J. Appl. Cryst.* **42**, 339–341.

[bb7] Gao, J., Zhang, F. & Zhang, X. (2024). *Adv. Sci.* **11**, 2400377.

[bb8] Graham, P. M., Pike, R. D., Sabat, M., Bailey, R. D. & Pennington, W. T. (2000). *Inorg. Chem.* **39**, 5121–5132. 10.1021/ic0005341 11233211

[bb9] Groom, C. R., Bruno, I. J., Lightfoot, M. P. & Ward, S. C. (2016). *Acta Cryst.* B**72**, 171–179. 10.1107/S2052520616003954 PMC4822653 27048719

[bb10] Harvey, P. D. & Knorr, M. (2016). *J. Inorg. Organomet. Polym.* **26**, 1174–1197.

[bb11] Hau, S. C. K., Yeung, M. C.-L., Yam, V. W.-W. & Mak, T. C. W. (2016). *J. Am. Chem. Soc.* **138**, 13732–13739. 10.1021/jacs.6b08674 27670800

[bb12] Johnsson, M., Törnroos, K. W., Mila, F. & Millet, P. (2000). *Chem. Mater.* **12**, 2853–2857.

[bb13] Kawamura, A., Greenwood, A. R., Filatov, A. S., Gallagher, A. T., Galli, G. & Anderson, J. S. (2017). *Inorg. Chem.* **56**, 3349–3356. 10.1021/acs.inorgchem.6b02883 28257185

[bb14] Liu, X. & Astruc, D. (2018). *Coord. Chem. Rev.* **359**, 112–126.

[bb15] Rao, V. M., Sathyanarayana, D. N. & Manohar, H. (1983). *J. Chem. Soc. Dalton Trans.* pp. 2167–2173.

[bb16] Rhodes, L. F., Huffman, J. C. & Caulton, K. G. (1985). *J. Am. Chem. Soc.* **107**, 1759–1760.

[bb17] Rigaku OD (2024). *CrysAlis PRO*. Rigaku Oxford Diffraction, Yarnton, England.

[bb18] Sculfort, S. & Braunstein, P. (2011). *Chem. Soc. Rev.* **40**, 2741–2760. 10.1039/c0cs00102c 21331417

[bb19] Sheldrick, G. M. (2015*a*). *Acta Cryst.* A**71**, 3–8.

[bb20] Sheldrick, G. M. (2015*b*). *Acta Cryst.* C**71**, 3–8.

[bb21] Silva, A. F., Calhau, I. B., Gomes, A. C., Valente, A. A., Gonçalves, I. S. & Pillinger, M. (2023). *ACS Biomater. Sci. Eng.* **9**, 1909–1918. 10.1021/acsbiomaterials.3c00140 PMC10091354 36996427

[bb22] Spek, A. L. (2015). *Acta Cryst.* C**71**, 9–18. 10.1107/S2053229614024929 25567569

[bb23] Spek, A. L. (2020). *Acta Cryst.* E**76**, 1–11. 10.1107/S2056989019016244 PMC6944088 31921444

[bb24] Tang, J. & Zhao, L. (2020). *Chem. Commun.* **56**, 1915–1925. 10.1039/c9cc09354k 31996886

[bb25] Troyano, J., Zamora, F. & Delgado, S. (2021). *Chem. Soc. Rev.* **50**, 4606–4628. 10.1039/d0cs01470b 33600546

[bb26] Yip, S.-K., Chan, C.-L., Lam, W. H., Cheung, K.-K. & Yam, V. W.-W. (2007). *Photochem. & Photobiol. Sci.* **6**, 365–371. 10.1039/b612334a 17404630

[bb27] Zhang, Y., Zhang, J., Li, Z., Qin, Z., Sharma, S. & Li, G. (2023*a*). *Commun. Chem.* **6**, 24. 10.1038/s42004-023-00817-5 PMC9908894 36755056

[bb28] Zhang, Y.-Z., Kong, X.-J., Zhou, W.-F., Li, C.-H., Hu, H., Hou, H., Liu, Z., Geng, L., Huang, H., Zhang, X., Zhang, D. & Li, J. (2023*b*). *Appl. Mater. Interfaces*, **15**, 4208–4215. 10.1021/acsami.2c19779 36625524

